# 3′‐UTR Hypermethylation and ABA Signaling Coordinate Transcriptional Activation of *SWEET14* for High Sugar Accumulation in Citrus

**DOI:** 10.1002/advs.78518

**Published:** 2026-10-31

**Authors:** Yike Zeng, Yue Wang, Yilei Wang, Haowei Chen, Qiyu Chen, Wei Xiao, Aoqing Zhang, Xin Jiang, Xiangming Shang, Zhicheng Yang, Xi Zeng, Wenqiang Hu, Chunlong Li, Ji‐Hong Liu

**Affiliations:** ^1^ National Key Laboratory for Germplasm Innovation and Utilization of Horticultural Crops College of Horticulture and Forestry Sciences Huazhong Agricultural University Wuhan Hubei China; ^2^ Hubei Hongshan Laboratory Wuhan Hubei China; ^3^ School of Horticulture Anhui Agricultural University Hefei Anhui China; ^4^ State Key Laboratory of Woody Oil Resources Utilization, Key Laboratory of Cultivation and Protection for Non‐Wood Forest Trees of the Ministry of Education Central South University of Forestry and Technology Changsha Hunan China

**Keywords:** chromatin loop, Citrus, *CliSWEET14*, DNA methylation, sucrose content, transcriptional cascade

## Abstract

Sugar accumulation is pivotal for fruit quality, yet the molecular regulation of this process remains elusive. By comparing transcriptomes of sweet lemon (*Citrus limon*, SL, high sugar level) and Eureka (EL, trace amount of sugar), we identified the sucrose transporter CliSWEET14 as a key driver of sugar accumulation. Methylome atlas showed that the 26‐bp sequence within 3′‐untranslated region (UTR) of *CliSWEET14‐SL* was substantially hypermethylated relative to *CliSWEET14‐EL*. Notably, the 3′‐UTRs of both genes formed chromatin loops with their native promoters. ClibHLH105 bound to unmethylated CACGAT sequence in the loop and repressed *CliSWEET14‐EL*. In contrast, hypermethylation of the CACGAT motif disrupted ClibHLH105 interaction and repression on *CliSWEET14‐SL*. Additionally, *CliSWEET14‐SL* was positively regulated by CliNAC56, which was further activated by CliABF3. Both CliNAC56 and CliABF3 regulated sucrose accumulation in a *CliSWEET14‐*dependent manner. Moreover, the regulatory cascade CliABF3‐CliNAC56‐*CliSWEET14* in SL was activated by ABA (abscisic acid) to a greater degree due to higher ABA level. Taken together, 3′‐UTR hypermethylation‐mediated relief of transcriptional repression and stronger activation by ABA signaling robustly boost *CliSWEET14* expression and sucrose accumulation. These findings substantially advance our understanding of sugar accumulation in fruit and provide novel epigenetic and transcriptional targets for improving fruit quality in citrus and beyond.

## Graphical abstract

Hypermethylation of *CliSWEET14* 3′‐UTR relieves ClibHLH105‐mediated transcriptional repression, together with ABA signaling‐mediated activation of the CliABF3‐CliNAC56 cascade, boosts *CliSWEET14* expression and sucrose accumulation in citrus.

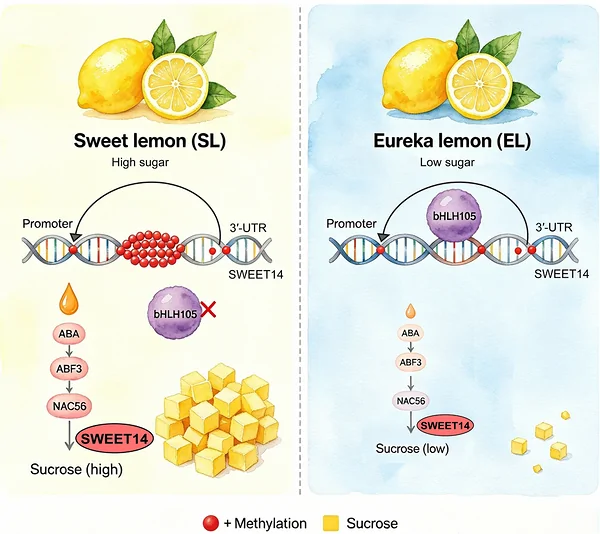

## 1 Introduction

Citrus represents one of the most economically, nutritionally, and culturally important fruit crops worldwide [1, 2, 3, 4]. As a major citrus group, lemons (*Citrus limon*) are widely appreciated for their characteristic acidity, aroma, and extensive application potential. Eureka lemon (EL) is one of the most commercially prevalent varieties with high acid content, but extremely low sugar levels [5, 6]. In contrast, sweet lemon (SL) exhibits drastically reduced citric acid accumulation and significantly elevated sugar content, rendering it well‐suited for fresh consumption [7]. Although sugar metabolism in fruit has been extensively investigated in diverse plant species, the molecular regulation of sugar accumulation remains far from being well exploited.

It has been well documented that sugar transport plays a critical role in the determination of sugar levels. Sugar transporters mediate transmembrane sugar flux, facilitating source‐to‐sink and cell‐to‐cell sugar allocation [8, 9]. They comprise sucrose transporters (SUT/SUC), monosaccharide transporters (MSTs), SWEETs (Sugars Will Eventually be Exported Transporters), and tonoplast‐localized TMTs [10, 11]. Among the sugar transporters, SWEETs play essential roles in transporting distinct sugar substrates to meet the needs of diverse developmental processes [10]. For example, AcSWEET10 in pineapple functions as a glucose transporter [12], whereas CmSWEET10 of melon serves as a sucrose transporter [13]. In *Arabidopsis*, AtSWEET11 and AtSWEET12 functioned as sucrose exporters, mediating the critical initial step of phloem loading [14]. Recently, *VvSWEET15* was shown to be implicated in the modulation of berry ripening through transporting hexose in grapevine [15]. Despite these findings, it is worth mentioning that molecular regulation of *SWEET* genes underlying fruit sugar accumulation remains elusive, and the associated signaling pathways warrant further comprehensive characterization.

DNA methylation, a major epigenetic modification occurring in CG, CHG, and CHH contexts, represents the most prevalent epigenetic mark and plays essential roles in regulating gene expression across diverse organisms [16, 17, 18]. Generally, DNA methylation in the promoter regions is associated with repression of gene transcription [19, 20]. For instance, hypermethylation at the promoter of target genes prevents efficient binding and activation of *OsERF3*, thereby attenuating disease resistance in rice [21]. Conversely, an increasing body of evidence has demonstrated that DNA methylation can also act as a positive regulator of gene expression [22]. A recent study in *Arabidopsis* reported that gene‐body methylation exerted a more positive effect on gene expression when transcriptional levels were relatively low [23]. In apple, methylation of a MITE (miniature inverted‐repeat transposable element) insertion was found to activate *MdRFNR1‐1* transcription and promote drought tolerance [24]. In sunflower, methylation of *HaWRKY6* promoter reshaped local chromatin architecture and enhanced *HaWRKY6* expression [25]. Despite the extensive illustration of methylation within promoters and gene bodies, the role of methylation within the 3′‐untranslated region (3′‐UTR) in the regulation of gene expression remains largely unknown.

Chromatin structure is fundamental to coordinating appropriate gene expression associated with diverse cellular processes [26]. Chromatin loops functionally tether distal regulatory elements and enable promoters and enhancers to exert long‐range effects on non‐adjacent genes, thereby modulating transcriptional activity [27]. Accumulating evidence indicates that three‐dimensional chromatin interactions between the 3′‐UTR and promoter regions play pivotal roles in the transcriptional regulation of plant genes [28, 29, 30]. For example, the promoter and 3′‐UTR of *Arabidopsis FLC* form a self‐loop that anchored the gene termini, a structure facilitating *FLC* expression [28, 31]. Similarly, chromatin looping between the proximal promoters and distal 3′‐UTR of *OsNCED3/5* directly modulated their transcription in rice [32]. Nevertheless, the involvement of chromatin looping in orchestrating gene expression networks associated with sugar accumulation remains largely unexplored.

The regulation of sugar accumulation in fruit is governed partly by the actions of plant hormones and transcriptional regulators [33, 34, 35, 36]. An increasing number of studies demonstrate that ABA orchestrates fruit sugar accumulation by regulating the expression of sugar transporter genes, thereby serving a pivotal role during fruit ripening. For instance, ABA induced *MdSWEET9b* expression to promote sucrose accumulation in apple [37]. Likewise, ABA was shown to mediate strawberry fruit ripening via modulation of the sugar transporter gene *STP13* [38]. However, the involved transcriptional cascade implicated in ABA‐mediated regulation of sugar accumulation remains to be unveiled. NAC transcription factors (TFs) are plant‐specific regulators harboring a conserved NAC domain [39, 40], and emerging evidence revealed that these NACs are tightly linked to ABA signaling and played vital roles in the regulatory network underlying fruit sugar metabolism. For example, *MdNAC1* was strongly ABA‐inducible through ABRE elements in its promoter [41], and *RcNAC091* exhibited a similar response to ABA induction [42]. Recently, *VvNAC72* was shown to function as a transcriptional activator that directly bound the *VvSWEET15* promoter to enhance hexose accumulation in grape [15]. Nevertheless, the molecular mechanism by which NACs bridge ABA signaling and sucrose accumulation remains poorly understood.

In our previous work, one of the sugar transporters, namely *CliSWEET14*, was found to exhibit substantially higher expression levels in SL than in EL [7]. However, the molecular basis underlying this difference in the two genotypes remains unclear. Herein, we characterized CliSWEET14 as a plasma membrane‐localized sucrose transporter. The promoter sequences of *CliSWEET14* from SL (*CliSWEET14‐SL*) and EL (*CliSWEET14‐EL*) were identical. Notably, the 3′‐UTR of both genes formed a chromatin loop with their promoters. Interestingly, hypermethylation of a CACGAT motif in the 3′‐UTR of SL disrupted binding of the transcriptional repressor ClibHLH105, thereby relieving its transcriptional repression on *CliSWEET14‐SL*, whereas *CliSWEET14‐EL* was repressed as the CACGAT motif was unmethylated. Additionally, *CliSWEET14* was demonstrated to be positively regulated by CliNAC56, which was further activated by CliABF3. The presence of higher ABA levels in SL led to more powerful transcription regulation of *CliSWEET14* by the hierarchical cascade CliABF3‐*CliNAC56*. Collectively, both relief of 3′‐UTR‐mediated transcriptional repression and stronger ABA‐induced transcriptional activation contribute to increased *CliSWEET14* expression and sucrose accumulation in SL. These findings provide novel insights into the molecular mechanisms underlying sugar accumulation and yield valuable target genes for genetic manipulation in the future.

## 2 Results

### 2.1 Assembly and Annotation of Sweet Lemon Genome Sequence

The genome of EL has been previously sequenced [7], providing insights into citrate accumulation, while that of SL remains uncharacterized, and the molecular basis underlying its sugar accumulation is also poorly understood. Therefore, we first sequenced the genome of SL by using Illumina, PacBio high‐fidelity long reads data (HiFi), and Oxford Nanopore (ONT) technologies (Table S1). Prior to genome assembly, k‐mer frequency analysis (k = 17) of the Illumina reads predicted a genome size of 320 Mb, with 3.1% heterozygosity and 57.34% repetitive sequences (Figure S1). For *de novo* genome assembly, 38.98 Gb of Nanopore reads and 26.12 Gb of PacBio HiFi reads were cleaned, assembled, and polished, yielding two haplotype‐resolved primary assemblies harboring contigs with N50 values of 36.99 Mb and 35.69 Mb, respectively. Integrating Hi‐C data, the assembly was scaffolded into 18 pseudochromosomes corresponding to the two haplotypes (Figure 1A). The finalized genome assembly was 659 Mb, consisting of Haplotype A (353 Mb, paternal haplotype) and Haplotype B (306 Mb, maternal haplotype), respectively. The genome assembly quality and completeness were evaluated using BUSCO and the long‐terminal repeat assembly index (LAI). BUSCO scores were 97.7% for Haplotype A and 98.9% for Haplotype B (Table 1), and the LAI score was higher than 16, indicating high assembly continuity. In addition, 34 telomeres were detected for the two haplotypes (Figure 1C). The majority (8) of chromosomes were assembled at telomere‐to‐telomere (T2T) level for each haplotype.

**FIGURE 1:**
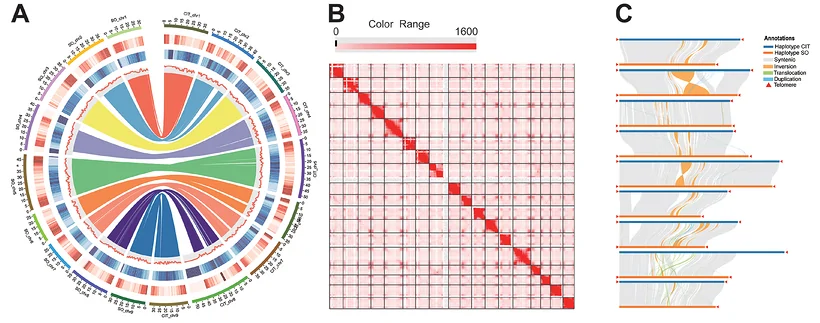
Genomic characteristics and structural variations in the genome of *Citrus limon*. (A) Circos plot illustrating major genomic features of the *Citrus limon* genome assembly. The outermost circle comprises 18 pseudochromosomes. Features depicted from the outermost to innermost circles include gene density, GC content and inter‐subgenome syntenic regions. All distributions are visualized within a window size of 1 Mb. (B) Hi‐C map showing genome‐wide interactions between chromosomes. (C) Structural variations between haplotypes. Comparison of structural variations between the citron Haplotype (CIT, used as the reference) and sour orange (SO) Haplotype of *C. limon* genomes. Triangles at both ends of the chromosome represent telomeres.

**TABLE 1:** Haplotype‐resolved chromosomal‐level assembly and annotation of lemon genome.

	Haplotype CIT	Haplotype SO
Assembly Size (Mb)	353	306
Contig N50 (Mb)	36.99	35.69
BUSCO Completeness of Assembly (%)	97.7	98.9
Number of Annotated Genes	23,532	22,155
LTR Assembly Index (LAI)	16.75	
QV	48.47	

Gene model prediction was performed using an integrated strategy combining *de novo* prediction, homology‐based prediction, and RNA‐seq data. In total, 23 532 and 22 155 protein‐coding genes were annotated in Haplotype A and Haplotype B, respectively. We subsequently evaluated the distribution density of genes and transposable elements (TEs) across the genome. TEs were predominantly concentrated in the central regions of chromosomes, where gene density was correspondingly low (Figure 1B).

### 2.2 Identification of *CliSWEET14* as a Critical Gene Differentially Expressed Between SL and EL During Fruit Development

To explore the differences between SL and EL, we first observed the fruit morphology of the two varieties. No obvious differences in fruit appearance were detected between them (Figure 2A). Then, we measured the levels of main soluble sugars, including sucrose, fructose, and glucose, in the fruits. During fruit development starting from 65 days after full blossom (DAFB), the contents of sucrose, fructose, and glucose in SL fruits showed a progressive increase. In contrast, the levels of these three sugars remained nearly constant throughout the entire developmental period of EL fruits. Notably, the sugar levels in SL were significantly higher than those of EL (Figure 2B).

**FIGURE 2:**
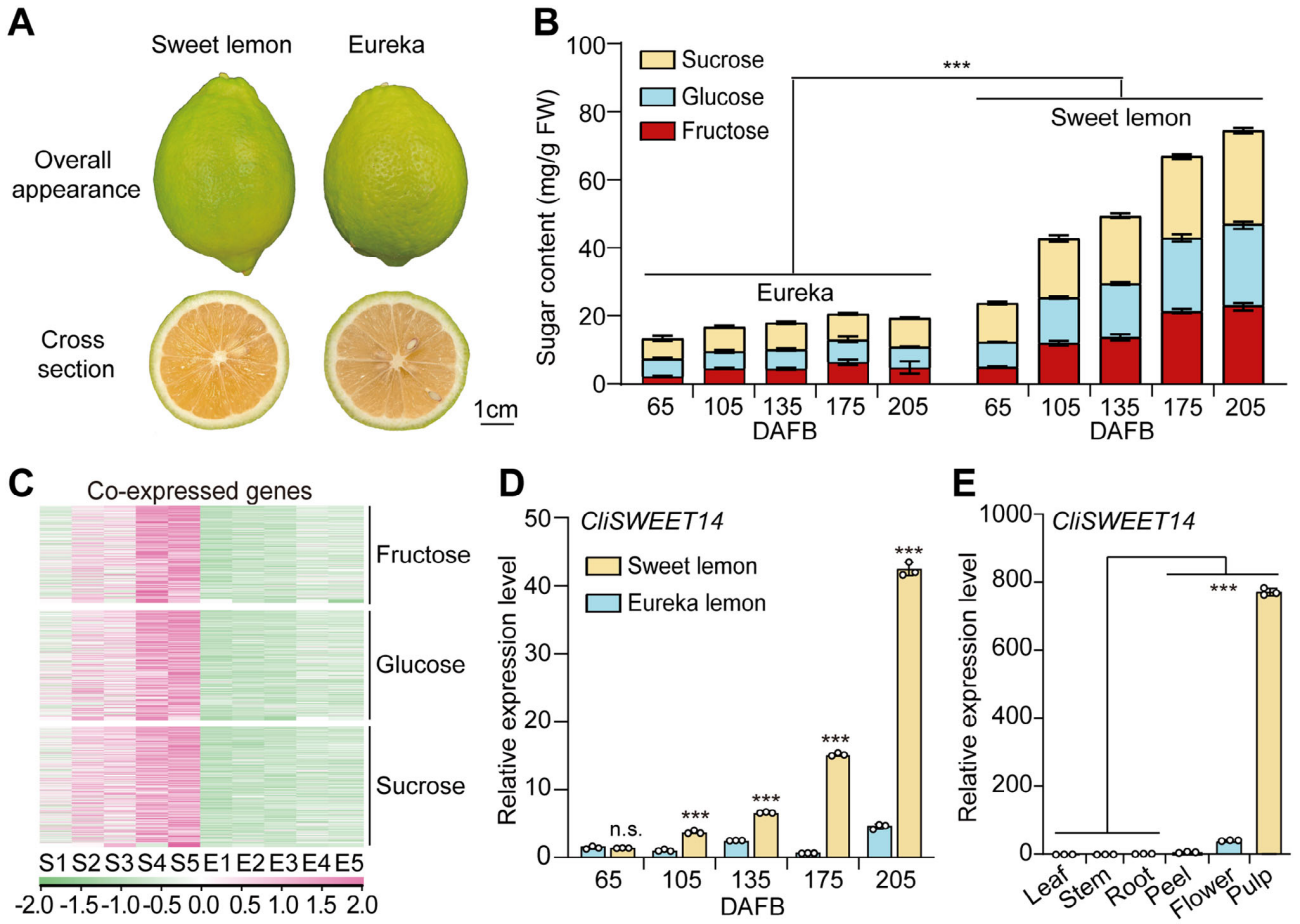
Soluble sugars contents and expression of associated genes in lemon. (A) Fruit morphology of Eureka and Sweet lemon at the ripening stage. Images were digitally extracted for comparison. Scale bar = 1 cm. (B) Contents of sucrose, fructose, and glucose in the fruit pulp of Eureka and Sweet lemon at 5 development stages. (C) Expression profiles of genes correlated with fructose, glucose, or sucrose contents during fruit development in Eureka and Sweet lemon. Gene sets were defined separately for each sugar using a Pearson correlation coefficient (PCC) threshold of ≥ 0.75, with overlapping membership allowed. (D) The expression level of *CliSWEET14* in the pulp of Eureka and Sweet lemon at five developmental stages. (E) The expression level of *CliSWEET14* in different tissues, including leaf, stem, flower, peel, pulp, and root, of sweet lemon. Error bars indicate ± *SD* (*n* = 3). Two‐tailed Student's *t*‐test was conducted for analyzing the significant difference (^***^p < 0.001). ns, no significance (*P* > 0.05). FW, fresh weight.

To further decipher the molecular basis underlying the marked differences in soluble sugar contents between the two lemon genotypes, RNA‐seq was conducted using fruits collected at the five time points. A total of 15 433 differentially expressed genes (DEGs) with FPKM > 2 in at least one time point were identified in SL relative to EL. Metabolic modules of the DEGs corresponding to sucrose, fructose, and glucose were constructed according to their accumulation patterns (Figure S2A). Pearson correlation coefficients (PCCs) between gene expression profiles and the contents of sucrose, fructose, and glucose were calculated separately so as to unravel genes associated with sugar accumulation in SL across the five developmental stages. As a result, we identified 348 DEGs that were correlated with at least one of the three sugars based on the PCC threshold of ≥ 0.75 (Figure 2C). Notably, most of the correlated genes were shared among the three sugars, whereas only a small group of the DEGs showed preferential correlation with an individual sugar (Figure S3). Gene Ontology (GO) analysis of the DEGs showed that terms of “Sugar mediated signaling pathway” and “Positive regulation of glucose‐mediated signaling pathway” were prominently enriched (Figure S2B). Among the DEGs correlated with soluble sugar accumulation, two sugar transporter genes, including *CliSWEET14* and *CliERD6L7*, draw our attention (Table S2). Both genes exhibited higher expression levels in SL than in EL, with the greater transcript abundance being observed for *CliSWEET14* throughout the fruit developmental process (Figure S4). Based on its transporter annotation, expression correlation with sugar levels, and relatively high transcript abundance, *CliSWEET14* was selected for further characterization.

At the early stage of fruit development, the expression level of *CliSWEET14* in SL (designated as *CliSWEET14*‐*SL* for the convenience of description) was comparable to that of EL (*CliSWEET14*‐*EL*). However, as fruit development progressed, the expression levels of *CliSWEET14‐EL* remained relatively stable, whereas transcript levels of *CliSWEET14*‐*SL* gradually increased until reaching the maximum level at the last time point. As a consequence, the RNA abundance of *CliSWEET14‐SL* was significantly higher than that of *CliSWEET14‐EL* during the late stages of fruit development (Figure 2D). Additionally, we found that the transcript level of *CliSWEET14‐SL* was predominantly detected in fruit pulp, followed by flowers, whereas it was barely detected in other tissues, including leaves, roots, stems, and peel (Figure 2E).

### 2.3 *CliSWEET14* is a Plasma Membrane‐Localized Sucrose Transporter

Given that the progressive upregulation of *CliSWEET14* coincided with elevated sugar accumulation in SL, we selected this gene for function analysis. Firstly, the spatial distribution of *CliSWEET14* transcripts in SL juice sacs was examined via in situ hybridization. The results showed that *CliSWEET14* was predominantly accumulated in the peripheral parenchyma layers (PPL) of juice sacs (Figure 3A). Subsequently, the subcellular localization of CliSWEET14 was determined using laser confocal microscopy of *Nicotiana benthamiana* leaf epidermal cells. Yellow fluorescent protein (YFP) signal was distributed in both cytoplasm and nucleus when expressing the control vector (35S: YFP), whereas expression of the 35S:CliSWEET14‐YFP led to fluorescence distribution in both the plasma membrane and secretory vesicles (Figure 3B).

**FIGURE 3:**
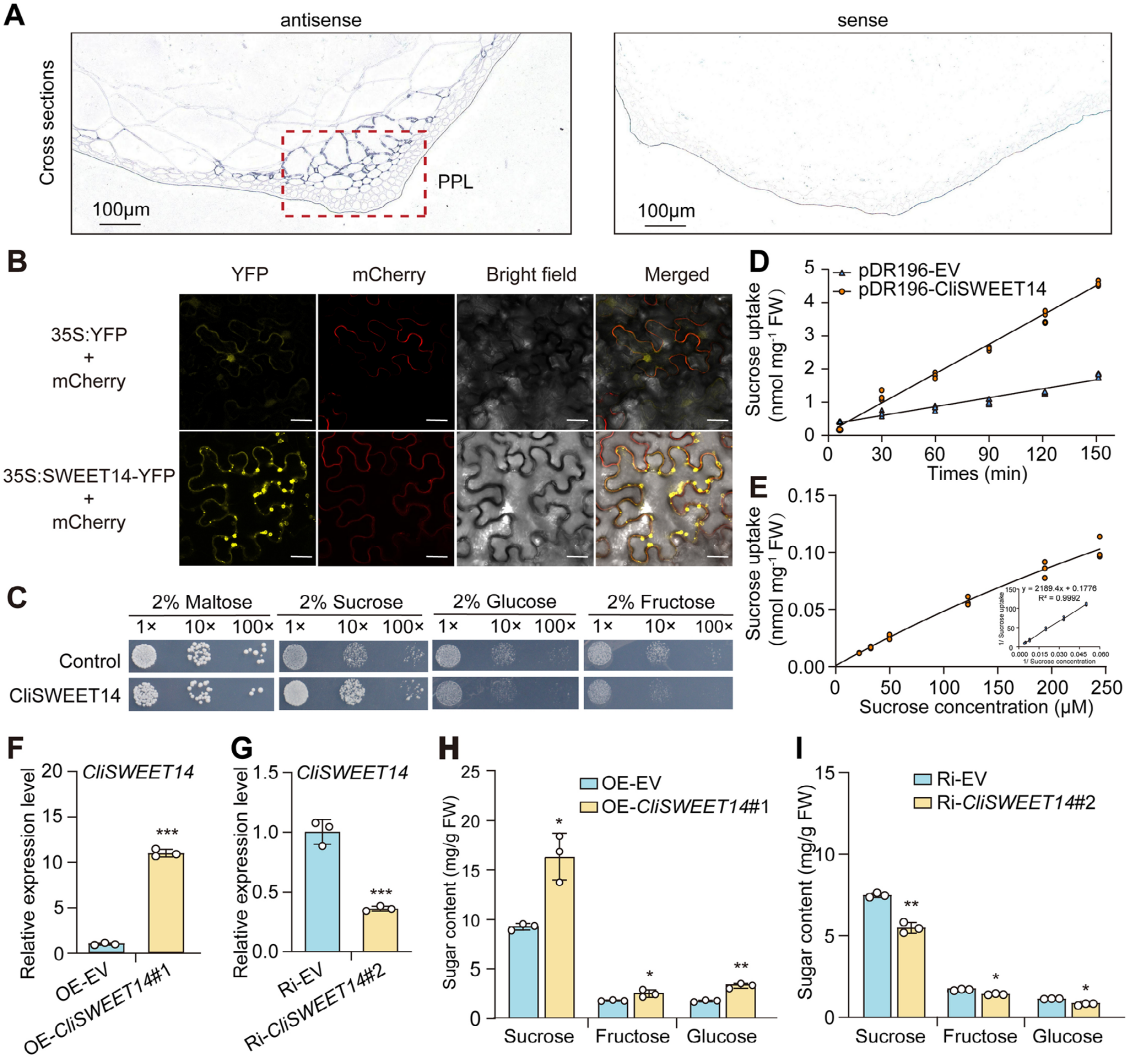
CliSWEET14 is a plasma membrane‐localized sucrose transporter. (A) Cell‐type‐specific in situ detection of *CliSWEET14* mRNA within transverse sections of sweet lemon juice sacs. The left and right images show hybridization with the digoxigenin‐labeled *CliSWEET14* antisense and sense probe, respectively. PPL, peripheral parenchyma layer. Scale bars = 100 µm. (B) The subcellular localization of CliSWEET14‐YFP in leaf epidermal cells of *N. benthamiana*. mCherry (mCherry‐AtCBL1‐OFP) works as a plasma membrane marker. Scales bar = 20 µm. (C) Growth of the sugar transporter‐deficient yeast (CSY4000) cells transformed with pDR196‐ CliSWEET14 or pDR196‐EV on Synthetic Dropout (SD)/‐U (Ura) medium added with maltose, glucose, fructose or sucrose. (D) Time‐course changes in the uptake rate of ^14^C‐sucrose by CSY4000 cells transformed with pDR196‐CliSWEET14 or pDR196‐EV. (E) Uptake rates of ^14^C‐sucrose in the CSY4000 cells expressing pDR196‐CliSWEET14 in the medium containing sucrose at different concentrations. The Lineweaver–Burk transform is performed on the data used to estimate Km. (F‐I) The expression level of *CliSWEET14* (F,G) and sugar contents (H,I) in transgenic citrus calli with overexpression (OE‐*CliSWEET14*) (F,H) or RNAi‐mediated knockdown (Ri‐*CliSWEET14*) (G,I) of *CliSWEET14*. OE‐EV and Ri‐EV are calli transformed with the empty vector of overexpression and RNAi constructs, respectively. Error bars indicate ± *SD* (*n* = 3). Two‐tailed Student's *t*‐test was conducted for analyzing the significant difference (^*^p < 0.05, ^**^p < 0.01, ^***^p < 0.001). ns, no significance, (*P* > 0.05). FW, fresh weight.

In order to characterize the transport properties of CliSWEET14, we cloned the 765‐bp coding sequences (CDS) of SL and EL, whose sequences are fully identical, into the pDR196 vector. The fusion construct (pDR196‐*CliSWEET14*) and empty vector (pDR196, used as a negative control) were separately transformed into a sugar transporter deficient yeast strain CSY4000, which cannot take up hexose or sucrose [43]. The yeast cells transformed with the fusion construct or the control vector grew well on the synthetic dropout (SD)/‐Ura (U) medium containing maltose, but failed to survive on the medium supplemented with glucose or fructose. In contrast, the yeast cells expressing pDR196‐CliSWEET14 could grow normally on the medium with sucrose as the sole carbon source, whereas the growth of negative control was suppressed (Figure 3C), indicating that CliSWEET14 could take up sucrose. To further determine the kinetics of *CliSWEET14*, we performed sugar uptake assays using ^14^C‐sucrose (^14^C‐Suc). As expected, the yeast cells transformed with pDR196‐*CliSWEET14* absorbed ^14^C‐Suc, whereas no uptake was observed in the negative control (Figure 3D). The *K_m_* of CliSWEET14 for sucrose uptake was 12.33 mm, with a maximum uptake rate (*V_max_*) of 5.63 nmol Suc min^−1^ mg^−1^ (Figure 3E). Collectively, these results demonstrate that CliSWEET14 was a sucrose transporter.

To assess the biological function of *CliSWEET14* in modulation of sucrose uptake, juice sacs of citrus fruit were transiently overexpressed with 35S: *CliSWEET14* or the empty vector (served as a control). The transgene overexpression was confirmed by quantitative real‐time PCR (qPCR) analysis (Figure S5A). Compared to the control, the juice sacs overexpressing 35S:*CliSWEET14* exhibited significantly higher sucrose content (Figure S5B). Furthermore, we generated transgenic citrus calli with overexpression (OE‐*CliSWEET14*) or RNA interference‐mediated knockdown of *CliSWEET14* (Ri‐*CliSWEET14*) via *Agrobacterium*‐mediated transformation (Figure 3F,G). Relative to the control (OE‐EV), the OE‐*CliSWEET14* line exhibited a significant increase in sucrose content, along with a slight elevation in fructose and glucose levels (Figure 3H). In contrast, silencing of *CliSWEET14* resulted in a significant reduction in sucrose levels, while fructose and glucose contents were also slightly but significantly reduced (Figure 3I). Collectively, these results confirmed that CliSWEET14 functions as a sucrose transporter and positively mediates sugar accumulation.

### 2.4 A 21‐bp Fragment in the 3′‐UTR of *CliSWEET14‐SL* was Hypermethylated

The finding that *CliSWEET14‐SL* transcript levels were significantly higher than those of *CliSWEET14*‐*EL* during the late stages of fruit development, concurrent with the higher sugar contents, prompted us to investigate the mechanism underlying this expression disparity. First, we compared the 2000‐bp sequences upstream of the transcription start site (TSS) of *CliSWEET14*‐*SL* and *CliSWEET14*‐*EL*, but did not detect any differences in transcription factor binding sites. We next profiled genome‐wide DNA methylation of both genes using SL and EL fruits at 65 and 135 DAFB, and found that the bisulfite conversion rates of the WGBS (whole‐genome bisulfite sequencing) libraries were higher than 99% (Table S3). The promoter regions and the gene bodies of *CliSWEET14*‐*SL* and *CliSWEET14*‐*EL* exhibited similar methylation patterns. However, a noticeable variation in DNA methylation was detected within their 3′‐UTR regions. The 26‐bp region located from +557 to +582 bp downstream of the transcriptional termination sites (TTS) of *CliSWEET14‐SL* displayed pronounced DNA hypermethylation at the two stages compared with the corresponding region in *CliSWEET14‐EL* (Figure 4A). Interestingly, the 26‐bp region of *CliSWEET14‐SL* harbored three cytosine residues with stable methylation in the CpG context, whereas such methylation was absent at the counterpart sequences of *CliSWEET14‐EL*. Differential cytosine methylation in this region was validated by both bisulfite sequencing (Figure 4B) and Chop‐PCR (Figure 4C) using methylation‐sensitive enzyme digestion. Of note, two obvious CpG methylation sites were noticeably detected within a 21‐bp region. Therefore, we focused our subsequent investigation on this 21‐bp region.

**FIGURE 4:**
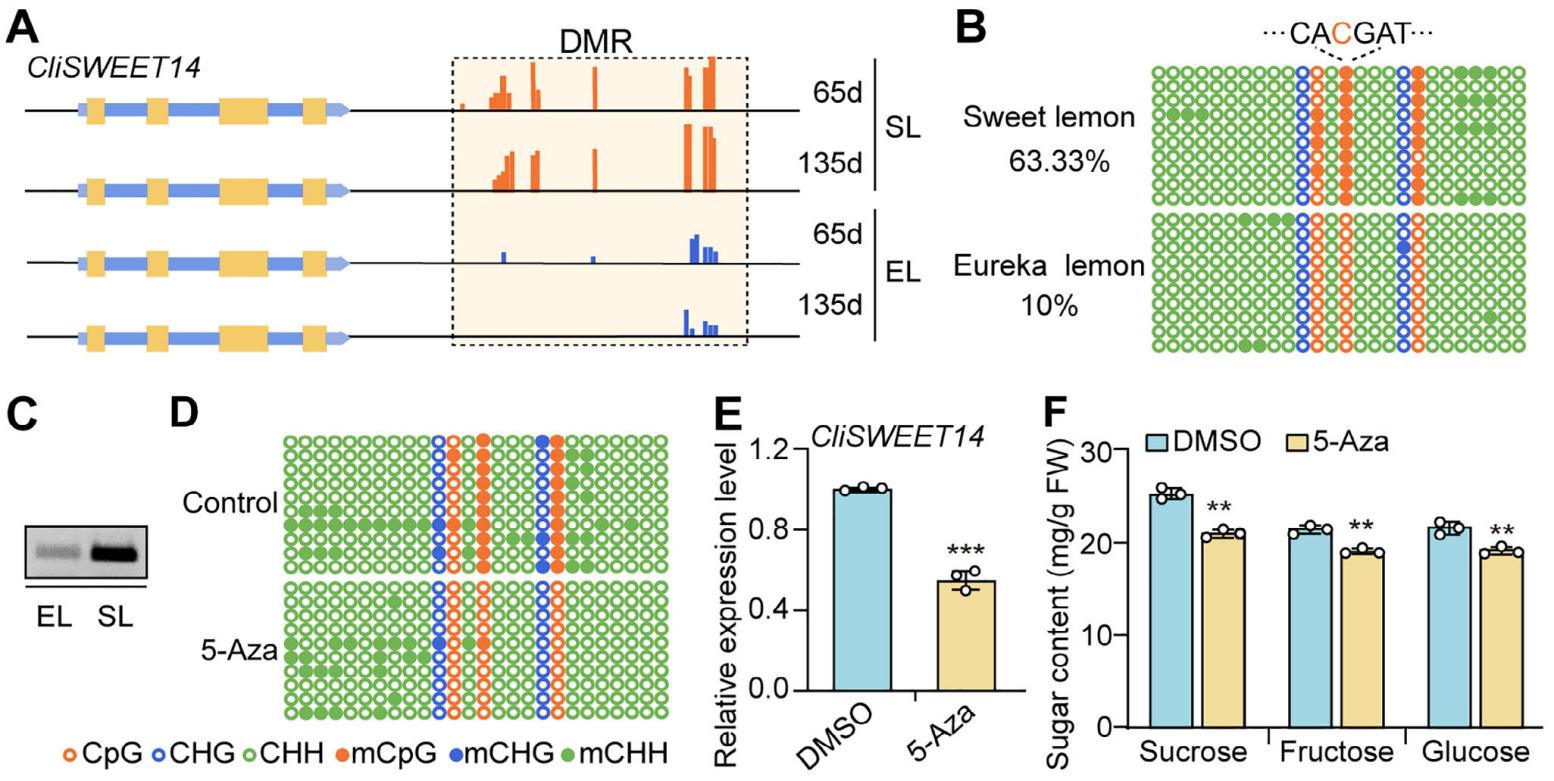
3'‐UTR hypermethylation affects the expression of *CliSWEET14*. (A) DNA‐methylation status of 3'‐UTRs of *CliSWEET14* from Eureka (EL) and Sweet lemon (SL) at two developmental stages based on whole‐genome bisulfite sequencing. (B,C) Variation of *CliSWEET14* DNA methylation in Eureka (EL) and sweet lemon (SL), as revealed by bisulfite sequencing (B) and Chop‐PCR (C). Methylation status of cytosines in 10 sequenced clones per amplicon was shown. The open and closed circles represent the nonmethylated and methylated cytosines, respectively. (D) 5‐Azacytidine treatment changed *CliSWEET14* DNA methylation in sweet lemon. Methylation status of cytosines in 10 sequenced clones per amplicon was shown. The open and closed circles represent the nonmethylated and methylated cytosines, respectively. (E,F) The expression levels of *CliSWEET14* (E) and sugar content (F) in sweet lemon fruit with or without 5‐Aza treatment. Error bars indicate ± *SD* (*n* = 3). Two‐tailed Student's *t*‐test was conducted for analyzing the significant difference (^**^p < 0.01, ^***^p < 0.001). FW, fresh weight.

Accumulating evidence has demonstrated that 3′‐UTRs may act as critical regulatory regions governing gene expression [28, 29, 30]. We therefore hypothesized that the differential methylation within the 3′‐UTR might modulate *CliSWEET14‐SL* expression. To test this hypothesis, we examined the transcript levels of *CliSWEET14‐SL* in SL fruits treated with 5‐Aza, an inhibitor of DNA methylation. 5‐Aza treatment markedly reduced the methylation level across the 21‐bp region of *CliSWEET14‐SL* (Figure 4D), accompanied by significant downregulation of *CliSWEET14‐SL* expression and reduced sucrose accumulation (Figure 4E,F). These results indicate that hypermethylation of the 21‐bp 3′‐UTR region may contribute to the transcriptional activation of *CliSWEET14‐SL*, thereby promoting sucrose accumulation in SL fruit.

### 2.5 Methylation Disrupted Transcriptional Repression of *CliSWEET14‐SL* by ClibHLH105

Previous studies have revealed that 3′‐UTR regions can form chromatin loops with cognate promoter regions, thereby modulating gene expression through chromatin interactions [30, 44]. To determine whether such a regulatory mode occurs at the 3′‐UTR of *CliSWEET14*, we analyzed the Hi‐C datasets of SL and EL fruit. Interestingly, it was found that the 500‐bp fragment downstream of the 21‐bp region within the 3′‐UTRs of both genotypes exhibited prominent chromatin interactions with their proximal promoters (Figure 5A). We further validated these long‐range spatial interactions by performing Chromosome Conformation Capture (3C) assays using *Dpn*II‐digested chromatin. Combining formaldehyde‐assisted isolation of regulatory elements with qPCR detection, we verified the occurrence of spatial contact between the promoters and 3′‐UTR regions (Figure 5B). As a result, folded chromatin conformations are formed at the *CliSWEET14* locus in both SL and EL, indicating that the 3′‐UTR of *CliSWEET14* formed a chromatin loop with its proximal promoter, which tethered the 21‐bp region to the promoters. Of note, one of the two hypermethylated cytosines is located in CACGAT, which resembles the N‐box (CACGAG) element that is recognized by bHLH TFs, and this motif is absent from the promoter region. We were curious to know whether the CACGAT‐containing sequences can be targeted by any bHLHs. To this end, we analyzed the transcriptome dataset and identified two bHLHs, *ClibHLH105* and *ClibHLH13*, that were expressed in both SL and EL (Figure S6). Then yeast one‐hybrid (Y1H) assays were conducted to examine the interaction between the two TFs and the CACGAT‐containing sequences from the 3′‐UTR physically associated with the promoter. The results showed that yeast cells transformed with prey of ClibHLH105, but not ClibHLH13, and the bait derived from the 21‐bp DNA fragment containing the CACGAT motif could survive in the selective medium added with AbA, implying that ClibHLH105 bind to the 21‐bp fragment harboring CACGAT, which is otherwise absent in the cognate promoter of *CliSWEET14* (Figure 6A). To clarify whether cytosine methylation interferes with ClibHLH105 binding to the CACGAT motif, we performed electrophoretic mobility shift assays (EMSA) using probes containing normal, mutated, or methylated (CAmCGAT) form of CACGAT sequence. EMSA showed that His‐ClibHLH105 bound well to the unmethylated normal CACGAT probe, while the binding was competed by an unlabeled probe. In contrast, methylation of the cytosine or mutation of the motif completely abolished the formation of the protein‐DNA complexes. However, binding affinity was not altered when the cytosine residues outside of the CACGAT motif were methylated (Figure 6B). To further test how ClibHLH105 transcriptionally regulated *CliSWEET14*, dual luciferase (LUC) transient expression assay was conducted. Co‐expression of the effector vector (SK‐bHLH105) and the reporter vector constructed using the 21‐bp fragment from the 3′‐UTR of *CliSWEET14‐EL* in *N. benthamiana* leaves led to a significant decrease in the promoter activity, as indicated by the LUC/REN ratio, relative to the control (Figure 6C). The quantitative measurement was further supported by LUC fluorescence imaging (Figure 6D). These results indicate that ClibHLH105 acted as a transcriptional repressor of *CliSWEET14‐EL* through targeting the normal CACGAT core sequence, while methylation of the cytosine residue in the CACGAT motif in *CliSWEET14‐SL* completely impaired the binding of ClibHLH105.

**FIGURE 5:**
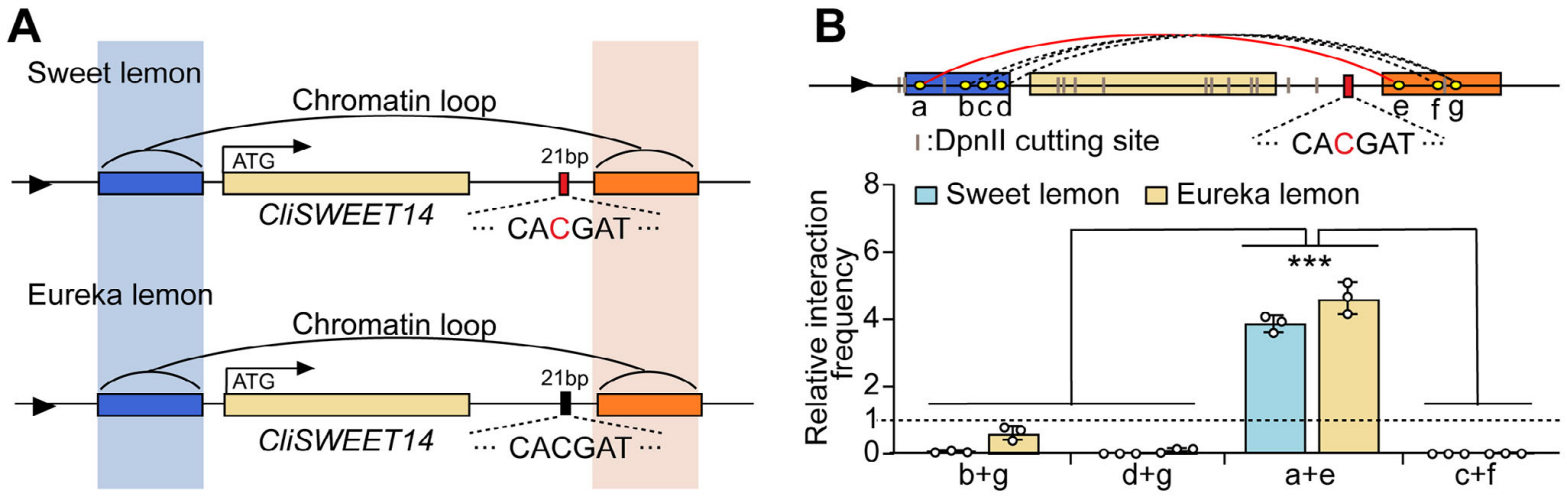
Chromatin interaction between the 3'‐UTR and its native promoter of *CliSWEET14*. (A) A schematic diagram showing the chromatin loop between *CliSWEET14* upstream promoter and the 3'‐UTR adjacent to CACGAT motif based on Hi‐C data from Eureka lemon and sweet lemon. (B) 3C‐PCR amplification of *CliSWEET14* chromatin DNA, with primers by the open circles indicated in the upper panel. Solid and dotted lines represent primer pairs tested for positive and negative 3C results, respectively. Error bars indicate ± *SD* (*n* = 3). Asterisks indicate that the value is significantly different from that of the control (^***^p < 0.001).

**FIGURE 6:**
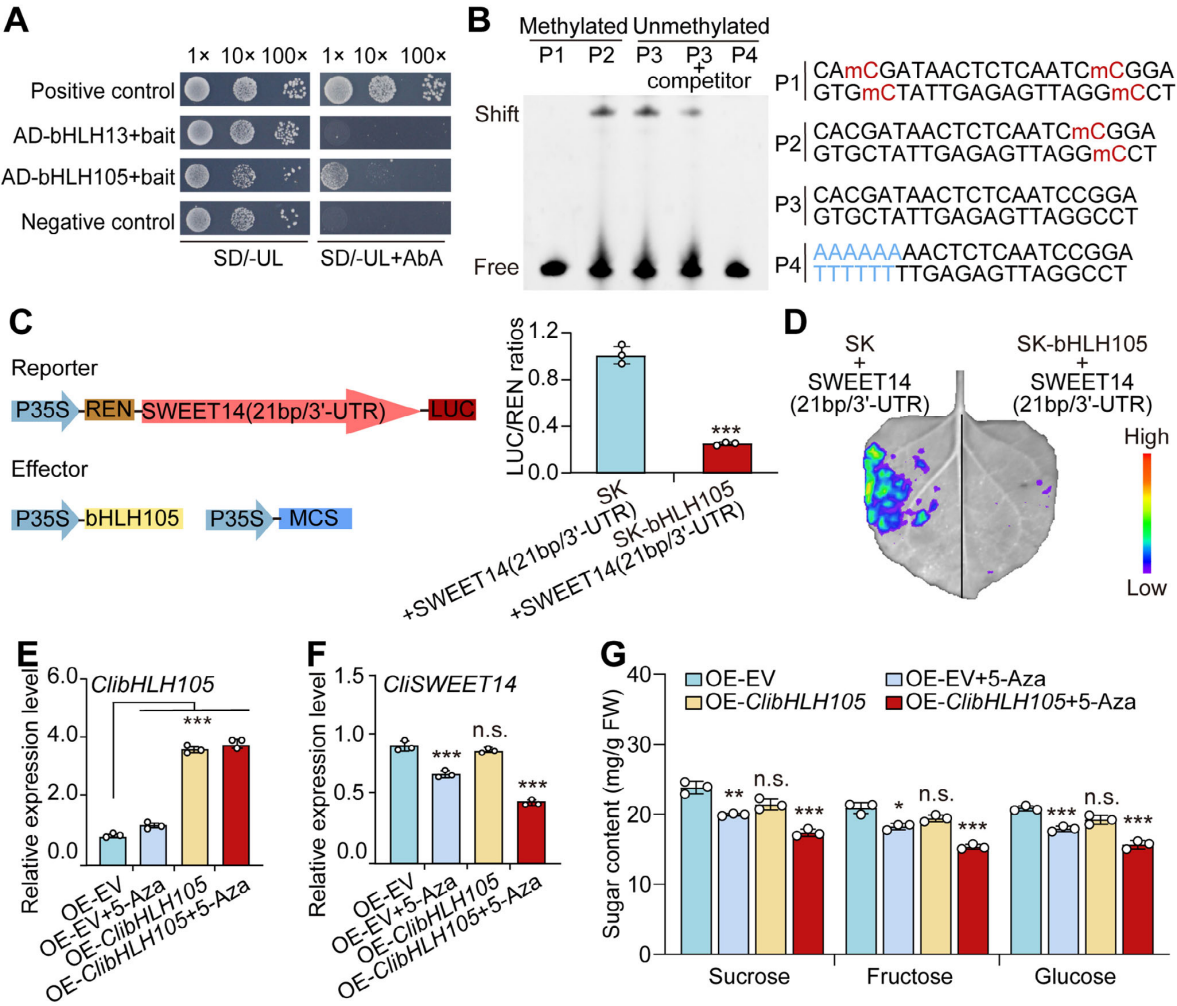
Methylation impairs the transcriptional repression of *CliSWEET14* by ClibHLH105. (A) Y1H assay identified the interaction between transcription factors and 44‐bp fragment containing the CACGAT motif from the 3'‐UTR of Sweet lemon *CliSWEET14*. Growth of yeast cells transformed with the bait constructed using hypermethylated 3'‐UTR and preys made using different transcription factors on the selective medium Synthetic Dropout (SD)/‐U (‐Ura)/‐L (‐Leu) with or without 200 mg/L AbA. pGADT7 + bait and pGADT7‐Rec‐p53 + p53‐AbAi were used as the negative and positive controls, respectively. (B) Electrophoretic mobility shift assay (EMSA) of HIS‐ClibHLH105 binding to the probe harboring a methylated cytosine in the ACGAT motif. Competitor probe is unlabeled DNA of P3 fragment. (C,D) Schematic diagrams of reporter and effector vectors for the dual‐luciferase (LUC) assays. LUC/REN ratios (C) and LUC bioluminescence imaging (D) in the *N. benthamiana* leaves transiently expressing the reporter constructed with the 44‐bp fragment of 3'‐UTR and the effector SK‐ClibHLH105. (E–G) The expression levels of *ClibHLH105* (E), *CliSWEET14* (F) and sugar content (G) in Sweet lemon juice sacs transiently overexpressing *ClibHLH105*, with or without 5‐Aza treatment. OE‐EV is calli transformed with the empty vector of overexpression construct. Error bars indicate ± *SD* (*n* = 3). Two‐tailed Student's *t*‐test was conducted for analyzing the significant difference (^*^p < 0.05, ^**^p < 0.01, ^***^p < 0.001). ns, no significance (*P* > 0.05). FW, fresh weight.

To verify the role of *ClibHLH105* in regulating sugar accumulation, we transiently overexpressed it in SL juice sacs (Figure S7). Surprisingly, overexpression of *ClibHLH105* alone had negligible effects on *CliSWEET14‐SL* expression and sucrose content. However, when 5‐Aza was applied, a moderate downregulation of *CliSWEET14‐SL* expression and reduction of sugar content were observed, with the effect being more profound in the *ClibHLH105*‐overexpressing juice sacs relative to those transformed with the empty vector (Figure 6E–G). These results demonstrate that ClibHLH105 plays a negative role in the regulation of *CliSWEET14‐SL* expression and sugar accumulation in a methylation‐dependent manner.

### 2.6 CliNAC56 Transcriptionally Activated *CliSWEET14* Expression

Except for the above‐mentioned hypermethylation‐mediated transcriptional repression release of *CliSWEET14*‐*SL*, we were curious to know whether *CliSWEET14‐SL* is regulated by other TFs to promote sucrose accumulation. To address this issue, we first analyzed the *CliSWEET14‐SL* promoter (pSWEET14) and identified multiple *cis*‐acting elements that are potentially recognized by various types of TFs (Figure S8). In order to decipher the candidate TFs, we used the 1442‐bp fragment upstream of the *CliSWEET14* TSS as a bait to screen an AD‐cDNA library. Four positive clones were sequenced (Table S4), including one candidate annotated as *CliNAC56*. It drew our attention as there was one NAC‐recognizing *cis*‐acting element (NACRS) in pSWEET14. Subcellular localization analysis showed that CliNAC56 was a nuclear‐localized protein (Figure 7A). To examine whether CliNAC56 exhibits transcriptional activation activity, a transactivation reporter assay was performed in yeast. The yeast cells grew well on the SD/‐T medium, whereas only those transformed with constructs containing full‐length or the C‐terminus of CliNAC56 grew normally on SD/‐Trp/‐His/‐Ade medium and exhibited α‐galactosidase activity (Figure 7B). These results demonstrate that CliNAC56 has transcription activation activity, with its activation domain located in the C‐terminal region.

**FIGURE 7:**
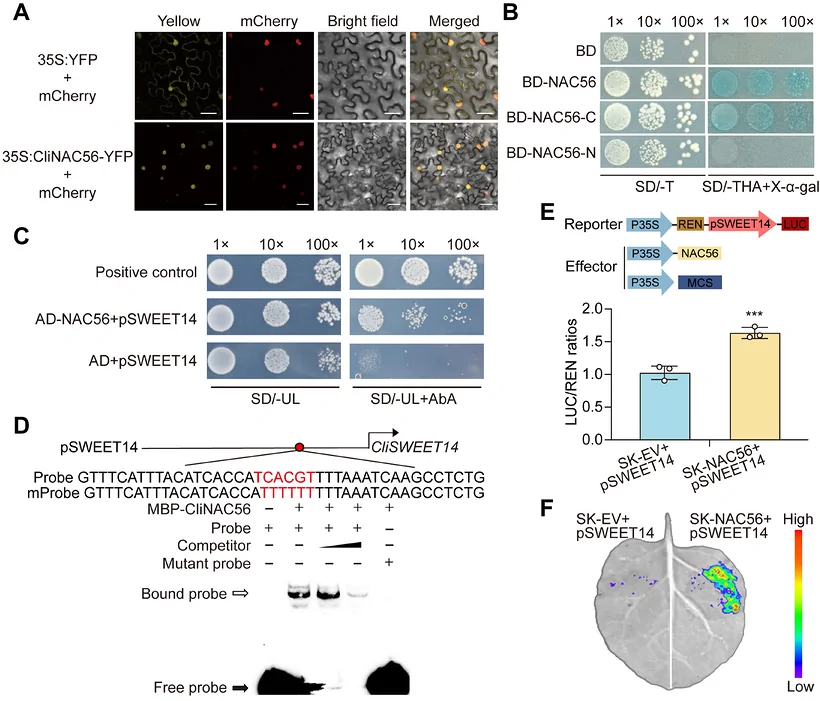
CliNAC56 activates *CliSWEET14* expression by binding to its promoter. (A) The subcellular localization of CliNAC56‐YFP in leaf epidermal cells of *N. benthamiana*. mCherry (mCherry‐VirD2NLS) works as a nucleus marker. Scale bars = 20 µm. (B) Growth of yeast (*Saccharomyces cerevisiae*) cells strain AH109 transformed with different vectors, using BD (pGBKT7) as a negative control, on Synthetic Dropout (SD)/‐T (Trp), and SD/‐T (Trp)/‐H (His) /‐A (Ade) added with X‐α‐gal. (C) Y1H assay identified binding of CliNAC56 to *CliSWEET14* promoter (pSWEET14). Growth of yeast cells transformed with the bait constructed using pSWEET14 and prey made from CliNAC56 on the selective medium Synthetic Dropout (SD)/‐U (‐Ura)/‐L (‐Leu) with or without 200 mg/L AbA. pGADT7 + pAbAi‐pSWEET14 and pGADT7‐Rec‐p53 + p53‐AbAi were used as the negative and positive controls, respectively. (D) EMSA confirming that CliNAC56 directly binds to the W‐box element of *CliSWEET14* promoter (pSWEET14). The protein‐DNA complex and free probe are shown by open and closed arrows, respectively. + and − represent the presence and absence, respectively, of the components shown on the top. Competitor, the same DNA fragment without biotin labeling. (E,F) Schematic diagrams of reporter and effector vectors for the dual‐luciferase (LUC) assays. LUC/REN ratios (E) and LUC bioluminescence imaging (F) in the *N. benthamiana* leaves transiently expressing the pSWEET14‐derived reporter and the effector SK‐NAC56. Error bars indicate ± *SD* (*n* = 3). Two‐tailed Student's *t*‐test was conducted for analyzing the significant difference (^***^p < 0.001).

To verify the interaction between CliNAC56 and pSWEET14, we first performed a point‐to‐point Y1H assay using CliNAC56 as a prey and pSWEET14 as a bait. Yeast cells co‐transformed with both prey and bait vectors grew well on the SD/‐UL added with AbA (Figure 7C), implying that CliNAC56 bound to the promoter of *CliSWEET14‐SL*. We further performed EMSA assay to confirm in vitro binding. Incubation of His‐CliNAC56 recombinant protein with the labeled probe harboring the NACRS element with a core sequence of CACG resulted in a band shift, which was weakened when competitive DNA was added. In addition, mutation of the CACG to TTTT completely abolished the band shift, implying that CliNAC56 interacted with pSWEET14 through the CACG motif (Figure 7D). To further test how CliNAC56 regulated *CliSWEET14* transcription, dual LUC transient expression assays were performed in *N. benthamiana* leaves. Co‐infiltration of the effector vector (SK‐CliNAC56) and the LUC reporter driven by pSWEET14 significantly increased LUC activity compared with the control (Figure 7E), in agreement with LUC fluorescence imaging (Figure 7F). Collectively, these results suggest that CliNAC56 binds to the *CliSWEET14* promoter at the NACRS motif and activates its expression.

### 2.7 CliNAC56 Enhanced Sugar Accumulation Through *CliSWEET14‐SL*

To verify the function of *CliNAC56* in sucrose regulation during lemon fruit development, we examined its expression pattern in both SL and EL. The transcript level of C*liNAC56* was reduced by nearly half at 105 DAFB and remained relatively constant throughout fruit development in EL. In contrast, C*liNAC56* expression increased continuously in SL and peaked at 175 DAFB, closely matching the expression pattern of *CliSWEET14‐SL* (Figure 8A). Moreover, *CliNAC56* exhibited predominant expression in pulp, with a spatial distribution profile comparable to that of *CliSWEET14*‐*SL* (Figure S9).

**FIGURE 8:**
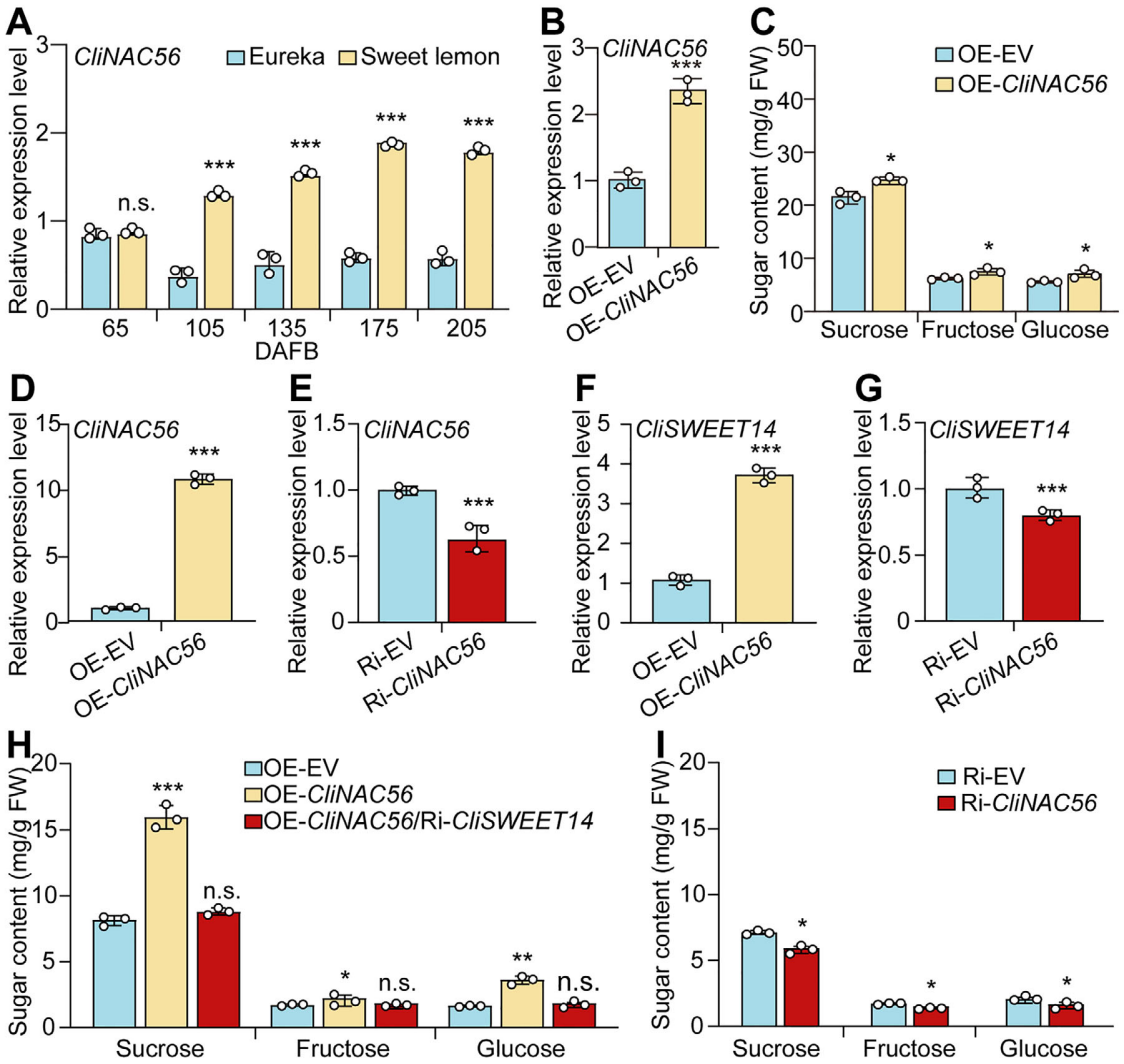
*CliNAC56* plays a positive role in the regulation of sugar accumulation. (A) The expression level of *CliNAC56* in the pulp of Eureka lemon and sweet lemon at five developmental stages. (B,C) The expression levels and sugar contents in transgenic citrus juice sacs transiently overexpressing *CliNAC56* (OE‐*CliNAC56*). (D–G) The expression levels of *CliNAC56* (D and E) and *CliSWEET14* (F and G) in transgenic citrus calli with overexpression (OE‐*CliNAC56*) or RNAi‐mediated knockdown (Ri‐*CliNAC56*) of *CliNAC56*. OE‐EV and Ri‐EV are calli transformed with the empty vector of overexpression and RNAi constructs, respectively. (H,I) The sugar contents in calli of OE‐EV, OE‐*CliNAC56*, OE‐*CliNAC56*/ Ri‐*CliSWEET14*, Ri‐EV, and Ri‐*CliNAC56*. OE‐*CliNAC56*/Ri‐*CliSWEET14* was the transgenic citrus calli with RNAi‐mediated transient repression of *CliSWEET14* in the calli of OE‐*CliNAC56*. Error bars indicate ± *SD* (*n* = 3). Two‐tailed Student's *t*‐test was conducted for analyzing the significant difference (^*^p < 0.05, ^**^p < 0.01, ^***^p < 0.001). ns, no significance (*P* > 0.05). FW, fresh weight.

We next performed functional assays to define the regulatory role of *CliNAC56* in sucrose accumulation. Transient overexpression of *CliNAC56* in juice sacs significantly promoted sucrose accumulation, while the levels of glucose and fructose were slightly but significantly increased (Figure 8B,C). We further generated transgenic citrus calli with overexpression (OE) and RNA interference‐mediated knockdown of *CliNAC56* via *Agrobacterium*‐mediated transformation (Figure 8D,E). Both *CliSWEET14* transcript levels and sucrose content were significantly elevated in OE‐*CliNAC56* calli (Figure 8F,H), but markedly reduced in Ri‐*CliNAC56* calli, in comparison with the control lines (OE‐EV and Ri‐EV) (Figure 8G,I).

To examine whether *CliNAC56* regulates sucrose levels in a *CliSWEET14‐SL* dependent manner, *CliSWEET14‐SL* was transiently silenced in OE‐*CliNAC56* transgenic calli (OE‐*CliNAC56*/Ri‐*CliSWEET14‐SL*). Sucrose content in the OE‐*CliNAC56*/Ri‐*CliSWEET14* calli was significantly reduced compared with that of the OE‐*CliNAC56* line, implying that silencing of *CliSWEET14‐SL* largely impaired the sucrose accumulation‐promoting effect conferred by *CliNAC56* overexpression (Figure 8H). These findings suggest that CliNAC56 acts genetically upstream of *CliSWEET14‐SL*, and enhances sucrose accumulation by regulating the expression of *CliSWEET14‐SL*.

### 2.8 *CliNAC56* was Transcriptionally Activated and Required by CliABF3 to Regulate Sucrose Accumulation

In non‐climacteric fruits, ABA acts as a positive regulator of sugar accumulation during fruit ripening [45, 46]. Accordingly, we profiled ABA metabolism‐related genes and measured endogenous ABA contents in SL and EL. The results revealed that expression levels of two ABA biosynthetic genes, *CliZEP* and *CliABA3*, were significantly higher in SL, whereas the ABA catabolic genes, *Cli707A2* and *Cli707A4*, were expressed at lower levels (Figure 9A). Consistently, ABA content in fruit pulp of SL was significantly higher than in EL (Figure 9B). These results suggest that SL produced more ABA relative to EL. Moreover, transcript levels of the ABA signaling genes, including *CliSnRK2.4*, *CliSnRK2.5*, *CliSnRK2.6*, and *CliSnRK2.10*, were also higher in SL (Figure 9A). This led us to assume that the ABA pathway in SL may contribute to sugar accumulation. To verify this assumption, we first treated citrus calli with ABA, and found that ABA treatment significantly induced the transcription of *CliNAC56* and *CliSWEET14* (Figure S10). Then, we examined whether and how ABA influenced the transcriptional activation ability of CliNAC56 to regulate *CliSWEET14*, by the dual LUC assay. Exogenous ABA treatment significantly elevated the promoter activity in the tobacco leaves expressing the effector (pNAC56: SK‐ NAC56) and the LUC reporter driven by pSWEET14 relative to those in the leaves expressing the same vectors under control treatment with water (Figure 9C,D). These results suggest that ABA promotes the transcriptional activation activity of CliNAC56 on *CliSWEET14*.

**FIGURE 9:**
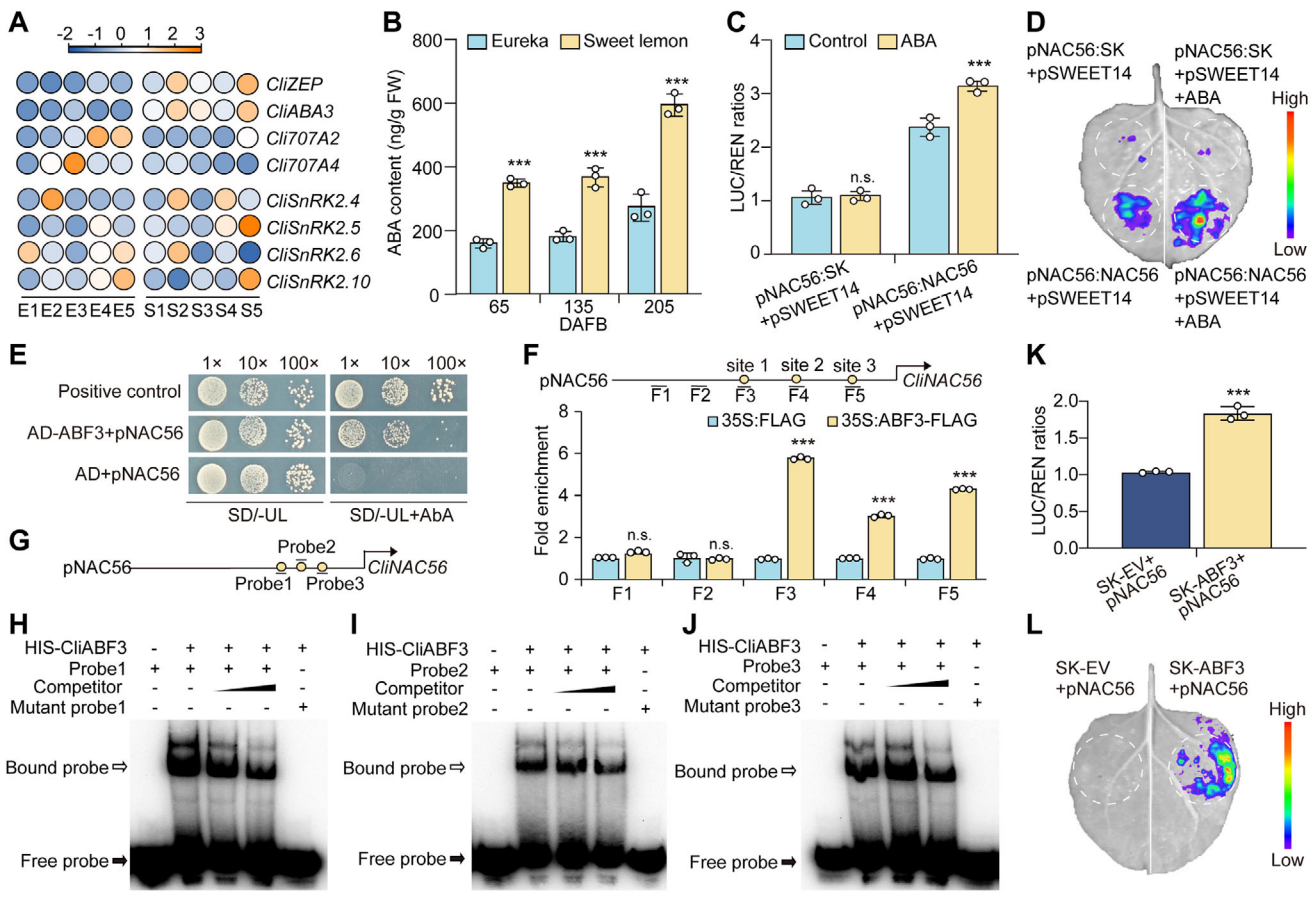
CliABF3 binds to the *CliNAC56* promoter and activates its expression. (A) Expression profile of genes involved in ABA metabolism and signaling. (B) Endogenous ABA contents in the pulp of Eureka lemon and sweet lemon at three developmental stages. (C,D) LUC/REN ratios (C) and LUC bioluminescence imaging (D) of *N. benthamiana* leaves co‐expressing effectors (pNAC56:SK or pNAC56:SK‐NAC56) with reporter vectors made from pSWEET14 in the presence or absence of ABA. (E) Y1H assay identified interactions of CliABF3 with *CliNAC56* promoter (pNAC56). Growth of yeast cells transformed with the bait constructed using pNAC56 and prey made from CliABF3 on the selective medium Synthetic Dropout (SD)/‐U (‐Ura)/‐L (‐Leu) with or without 200 mg/L AbA. pGADT7 + pAbAi‐pNAC56 and pGADT7‐Rec‐p53 + p53‐AbAi were used as the negative and positive controls, respectively. (F) ChIP‐qPCR assay reveals enrichment of CliABF3 in the promoter of *CliNAC56* (pNAC56) using primers designed for the five regions, F1‐F5. Transgenic calli overexpressing 35S: CliABF3‐FLAG was used for ChIP‐qPCR, using 35S: FLAG as a control. (G–J) EMSA confirming that CliABF3 directly binds to the promoter of *CliNAC56* (pNAC56, (G) containing three ABRE elements, which are located in the fragments used for synthesis of Probe 1 (H), Probe 2 (I), and Probe 3 (J). The protein‐DNA complex and free probe are shown by open and closed arrows, respectively. + and—represent the presence and absence, respectively, of the corresponding components. Competitor, the same DNA fragment without biotin labeling. (K,L) LUC/REN ratios (K) and LUC bioluminescence imaging (L) in the *N. benthamiana* leaves transiently expressing the pNAC56‐derived reporter and the effectors containing SK‐ABF3. Error bars indicate ± *SD* (*n* = 3). Two‐tailed Student's *t*‐test was conducted for analyzing the significant difference (^***^p < 0.001). ns, no significance (*p* > 0.05).

To further elucidate the interplay between ABA signaling and CliNAC56‐*CliSWEET14* module, we analyzed the 2000‐bp promoter sequences of *CliNAC56* and *CliSWEET14*. Three ABA‐responsive elements (ABREs, ACGTG) were identified in the *CliNAC56* promoter, whereas no ABREs were found in the promoter of *CliSWEET14* (Figure S8; Figure S11). We therefore examined the transcript levels of *ABF* family genes in both SL and EL, and found that *CliABF3* showed considerable transcriptional abundance (Figure S12). Subcellular localization analysis showed that CliABF3 was a nuclear‐localized protein. In addition, CliABF3 was found to possess the transcription activation activity, with the activation domain being located in the C‐terminal region (Figure S13). Subsequently, Y1H showed that CliABF3 bound to the *CliNAC56* promoter (pNAC56) (Figure 9E). In addition, ChIP‐qPCR assays demonstrated that CliABF3 was dramatically enriched in the three promoter fragments harboring the ABRE core motifs (Figure 9F). Furthermore, EMSA assays using the probes containing the three ABRE elements demonstrated that His‐CliABF3 recombinant protein directly targeted the three ACGTG sequences, and this interaction was completely abolished when each of the motifs was mutated (Figure 9G–J). Finally, dual LUC assays indicated that CliABF3 functions as a transcriptional activator of *CliNAC56* (Figure 9K,L). In summary, these results show that CliABF3 bound to the promoter of *CliNAC56* through the ABRE elements and activated its expression.

Efforts were made to elucidate the function of *CliABF3* in modulation of sugar accumulation by generating transgenic citrus calli with overexpression (OE‐*CliABF3*) and RNA interference‐mediated knockdown (Ri‐*CliABF3*) via *Agrobacterium*‐mediated transformation. Compared with the control line (OE‐EV), overexpression of *CliABF3* significantly elevated the expression levels of *CliNAC56* and *CliSWEET14* (Figure 10A–C), along with a marked increase in sucrose content (Figure 10D). Conversely, knockdown of *CliABF3* led to a prominent reduction in the expression of *CliNAC56* and *CliSWEET14* (Figure 10E–G), as well as a noticeable decrease in sucrose content relative to the control (Figure 10H), indicating that ABF3 plays a positive function in regulation of sucrose accumulation. To further investigate the genetic relationship between *CliABF3*, *CliNAC56*, and *CliSWEET14*, we transiently expressed the RNAi constructs of *CliNAC56* (Ri‐*CliNAC56*) or *CliSWEET14* (Ri‐*CliSWEET14‐SL*) in the *CliABF3‐*overexpressing calli (OE‐*CliABF3*), generating OE‐*CliABF3*/Ri‐*CliNAC56* and OE‐*CliABF3*/Ri‐*CliSWEET14* calli. As shown in Figure 10D, the sucrose level in the OE‐*CliABF3*/Ri‐*CliNAC56* and OE‐CliABF3/Ri‐*CliSWEET14‐SL* were significantly decreased relative to that of OE‐*CliABF3*, implying that knockdown of *CliNAC56* or *CliSWEET14‐SL* attenuated the sucrose‐inducing effect of *CliABF3* and that *CliABF3* functioned to regulate sucrose accumulation in a CliNAC56‐*CliSWEET14‐SL* dependent manner.

**FIGURE 10:**
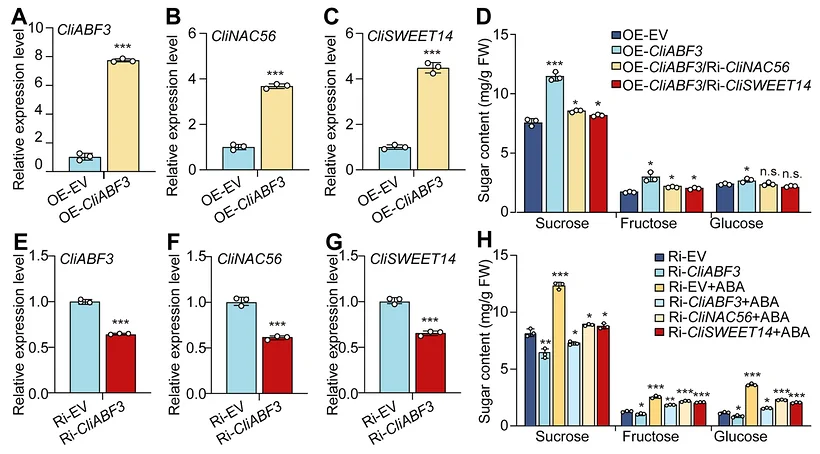
ABA signaling and *CliABF3* positively regulate sugar accumulation. (A–C) The expression level of *CliABF3* (A), *CliNAC56* (B), and *CliSWEET14* (C) in transgenic citrus calli overexpressing *CliABF3* (OE‐*CliABF3*). OE‐EV is calli transformed with the overexpression empty vector. (D) The sugar contents in the calli of OE‐EV, OE‐*CliABF3*, OE‐*CliABF3*/Ri‐*CliNAC56*, OE‐*CliABF3*/Ri‐*CliSWEET14*. OE‐*CliABF3*/Ri‐*CliNAC56* and OE‐*CliABF3*/Ri‐*CliSWEET14* were the transgenic citrus calli with RNAi‐mediated transient repression of *CliNAC56* or *CliSWEET14* in the calli of OE‐*CliABF3*. (E–G) The expression level of *CliABF3* (E), *CliNAC56* (F), and *CliSWEET14* (G) in transgenic citrus calli with RNAi‐mediated knockdown (Ri‐*CliABF3*) of *CliABF3*. Ri‐EV is calli transformed with the empty vector of RNAi. (H) The sugar contents in calli of Ri‐EV, Ri‐*CliABF3*, Ri‐*CliNAC56*, Ri‐*CliSWEET14* with or without ABA treatment. Error bars indicate ± SD (*n* = 3). Two‐tailed Student's *t*‐test was conducted for analyzing the significant difference (^*^ *p* < 0.05, ^**^ *p* < 0.01, ^***^ *p* < 0.001). ns, no significance (*P* > 0.05). FW, fresh weight.

To dissect how differences in ABA levels influenced sucrose accumulation mediated by the CliABF3‐*CliNAC56*‐*CliSWEET14* module, we first subjected transgenic citrus calli to ABA treatment. The results showed that, compared with the control (Ri‐EV), ABA treatment (Ri‐EV+ABA) significantly promoted sucrose accumulation in citrus calli (Figure 10H). Conversely, silencing each of *CliABF3*, *CliNAC56*, and *CliSWEET14* prominently attenuated the ABA‐induced sucrose accumulation to various degrees (Figure 10H). Collectively, these results demonstrate that ABA plays a critical regulatory role in sucrose accumulation mediated by the CliABF3‐*CliNAC56*‐*CliSWEET14* module. These findings imply that, relative to EL, the higher ABA level in SL positively regulates sucrose accumulation by amplifying the activation of this module.

## 3 Discussion

Sugar accumulation in fleshy fruits is a complex and sophisticated trait that depends on the coordinated balance of synthesis, degradation, and transport, and is also subjected to modulation by developmental, environmental, and hormonal signals [15, 47]. Among these, the transport of sugars from source to sink organs like fruit is mediated by sugar transporters, represents a critical rate‐limiting step that directly establishes the sugar accumulation capacity of the fruit [13, 48]. In this study, we identified the plasma membrane‐localized sucrose transporter CliSWEET14 as a central regulator of sugar accumulation in lemon fruit by dissecting the haplotype‐resolved genome of SL and performing comparative analysis of transcriptome atlas between SL and EL, two lemon genotypes with contrasting level of sugar. We further elucidated the synergistic regulation of *CliSWEET14* expression by epigenetic modifications and ABA signaling, which extends the functional understanding of the SWEET family in quality formation in non‐climacteric fruits. Our findings provide a theoretical basis and valuable gene resources for the improvement of sugar content in citrus and other fruit crops.

SWEET transporters exhibit diverse patterns of transmembrane topology and subcellular localization, thereby influencing processes such as phloem loading, nectar secretion, stress responses, and plant‐pathogen interactions [49, 50, 51]. A range of SWEET family members have been functionally characterized as key regulators of sugar accumulation in various plant species. For instance, AtSWEET9 and AtSWEET11/12, localized in the plasma membrane, have been demonstrated to function as sucrose transporters in A. *thaliana* [51, 52]. In addition, SlSWEET15 from tomato and ClSWEET3 from watermelon were shown to localize to the plasma membrane and act as transporters of sucrose and hexose, respectively [53, 54]. CitSWEET6 has been characterized as a plasma membrane‐localizing fructose transporter implicated in the elevation of fructose levels in citrus fruit [55]. In this study, we identified CliSWEET14 as a sugar transporter specifically expressed in the pulp of high‐sugar‐accumulating SL. CliSWEET14 was shown to localize to the plasma membrane and secretory vesicles, implying that it may dynamically regulate plasma membrane transport activity through vesicle trafficking. By conducting transgenic assays for functional characterization, we demonstrated that CliSWEET14 functions as a transporter of sucrose and plays a key role in promoting sucrose accumulation. Our results, together with those of earlier studies, seem to suggest that various SWEET members contribute to the transport of different sugars, and that their roles are versatile and may vary depending on the plant species, tissues, or organs examined. Notably, differences in the sugar profiles were detected between the callus and juice sacs in the assays. This may be ascribed to supplementation of sucrose alone, rather than glucose and fructose, in the callus culture system, while the juice sacs per se contained all tested sugars. In this regard, juice sacs are particularly suitable for elucidating the function of candidate genes involved in sugar accumulation through transient assays.

DNA methylation is a well‐characterized epigenetic modification that plays a pivotal role in regulating gene expression [56, 57]. For instance, cold stress triggered the loss of DNA methylation of the *ACT1* promoter and facilitated its binding by Dof1, leading to activated *ACT1* expression and enhanced cold tolerance in rice [58]. Similarly, in vivo binding of RIN to its target gene promoters was directly impaired due to hypermethylation of the sequences in the promoters [59]. These results illustrate that methylation of the *cis*‐acting elements in the promoters can directly modulate their interaction and binding with relevant transcription factors, thus influencing the expression of genes involve in diverse biological processes. On the other hand, chromatin structure has been also recognized as a critical factor in regulating gene expression and orchestrating a variety of cellular processes [26]. In plants, three‐dimensional chromatin contacts have emerged as valuable modulators of transcriptional regulation, as manifested by gene loop‐mediated impact on *FLC* expression and flowering time [27, 31, 60, 61]. However, the role of 3′‐UTR methylation, in synergy with formation of chromatin loop, in regulation of gene expression remains poorly understood hitherto. Interestingly, the methylation level of a 21‐bp fragment in the 3′‐UTR was positively correlated with transcript level of *CliSWEET14* in SL relative to EL. Mechanistically, we demonstrated that 3′‐UTRs from both genotypes could form a chromatin loop with their intrinsic promoters. As a result, the CACGAT core sequence in the 21‐bp fragment from EL was successfully recognized by ClibHLH105, which acted as a transcriptional repressor of *CliSWEET14*. By contrast, hypermethylation of the same motif in SL impaired the binding of ClibHLH105 and relieved its repressive effects on *CliSWEET14*, which was consequently activated and executed its normal function in facilitating sucrose transport. Thus, 3′‐UTR hypermethylation exerts a positive regulatory role in activating gene expression by preventing interaction with a transcriptional repressor, a mechanism that expands our understanding of the diverse functions of DNA methylation beyond the conventional view of it as a repressive mark. Together with previous reports in other systems, our findings suggest that methylation of *cis*‐acting elements from both promoter regions and 3’‐UTR may represent a universal paradigm for modulating gene expression across diverse biological contexts. In addition, our findings expand earlier framework through illustrating a fruit quality‐centered regulatory scenario mediated by formation of a chromatin loop. Whether such regulatory machinery, orchestrated by both chromatin loop and 3’‐UTR methylation, also operate in a broader physiological and developmental contexts beyond the one studied here remains an open question that warrants further extensive investigation in the future.

Transcriptional regulation of *SWEETs* has been studied in the context of stress responses, development, and fruit quality formation. For example, *ZjSWEET11* and *ZjSWEET18* of Chinese jujube were shown to be regulated by the ABA‐responsive transcription factor ZjABF1, leading to sugar accumulation and enhanced drought and salt tolerance [62]. Likewise, *VvSWEET15* from grape was activated by VvNAC72 to promote hexose accumulation during the course of berry véraison [15]. In the present study, we showed that *CliSWEET14* was transcriptionally activated by the NAC transcription factor CliNAC56 that was in turn positively regulated by CliABF3, thereby establishing a regulatory cascade of CliABF3‐*CliNAC56*‐*CliSWEET14*. Given that ABF3 is a crucial transcription factor in ABA signaling it is reasonable to assume that this pathway is largely integrated into the transcriptional regulation of *CliSWEET14*. Since the fruit of SL produced more ABA relative to EL, it is plausible that the CliABF3‐*CliNAC56*‐*CliSWEET14* molecular module was mobilized to a greater extent in the former, which may partially explain the accumulation of sucrose in SL when compared with EL. Of note, the change in the contents of ABA was incomparable or unsynchronous to that of sugars when the SL fruit developed over time, as evidenced by a more pronounced elevation in sugars relative to a mild alteration of ABA. Such discrepancy might arise because ABA largely functions as a key signaling molecule, and even subtle fluctuations in its level may trigger extensive transcriptional or metabolic reprogramming. However, the possibility that the sugars may exert feedback repression on the ABA accumulation cannot be fully ruled out. However, the interplay between CliABF3‐*CliNAC56‐* mediated transcriptional regulation and the chromatin loop‐mediated epigenetic modification of *CliSWEET14* remains to be elucidated. In particular, whether these two layers of regulation act independently or synergistically, and whether they act simultaneously or in a sequential manner, to promote sugar accumulation warrants further investigation.

In conclusion, we propose an integrated regulatory framework in which DNA methylation‐dependent chromatin architecture modification and ABA signaling jointly regulate *CliSWEET14* expression and sucrose accumulation (Figure 11). On the one hand, the formation of a chromatin loop and the presence or absence of 3′‐UTR methylation influence ClibHLH105‐mediated transcriptional repression of *CliSWEET14*. In the low‐sugar genotype (Eureka), ClibHLH105 successfully binds to the unmethylated CACGAT motif and thereby represses *CliSWEET14* expression, whereas hypermethylation of the same motif in the high‐sugar genotype (Sweet lemon) prevents ClibHLH105 binding, thereby relieving its repressive capacity and leading to activation of *CliSWEET14*. On the other hand, the higher ABA content in sweet lemon triggers the ABA signaling and thus activates the regulatory cascade of CliABF3‐*CliNAC56*‐*CliSWEET14* more robustly, thereby upregulating *CliSWEET14* more strongly. Together, these two tiers of regulation act to boost *CliSWEET14* expression, leading to intensive sucrose accumulation. Taken together, we dissect a novel mechanism whereby unique patterns of epigenetic modification and phytohormone signaling account for genotypic differences in transcriptional regulation of *CliSWEET14*. These findings advance our understanding of the molecular regulation of sugar accumulation in fruit and offer valuable targets for breeding varieties with optimized sugar content.

**FIGURE 11:**
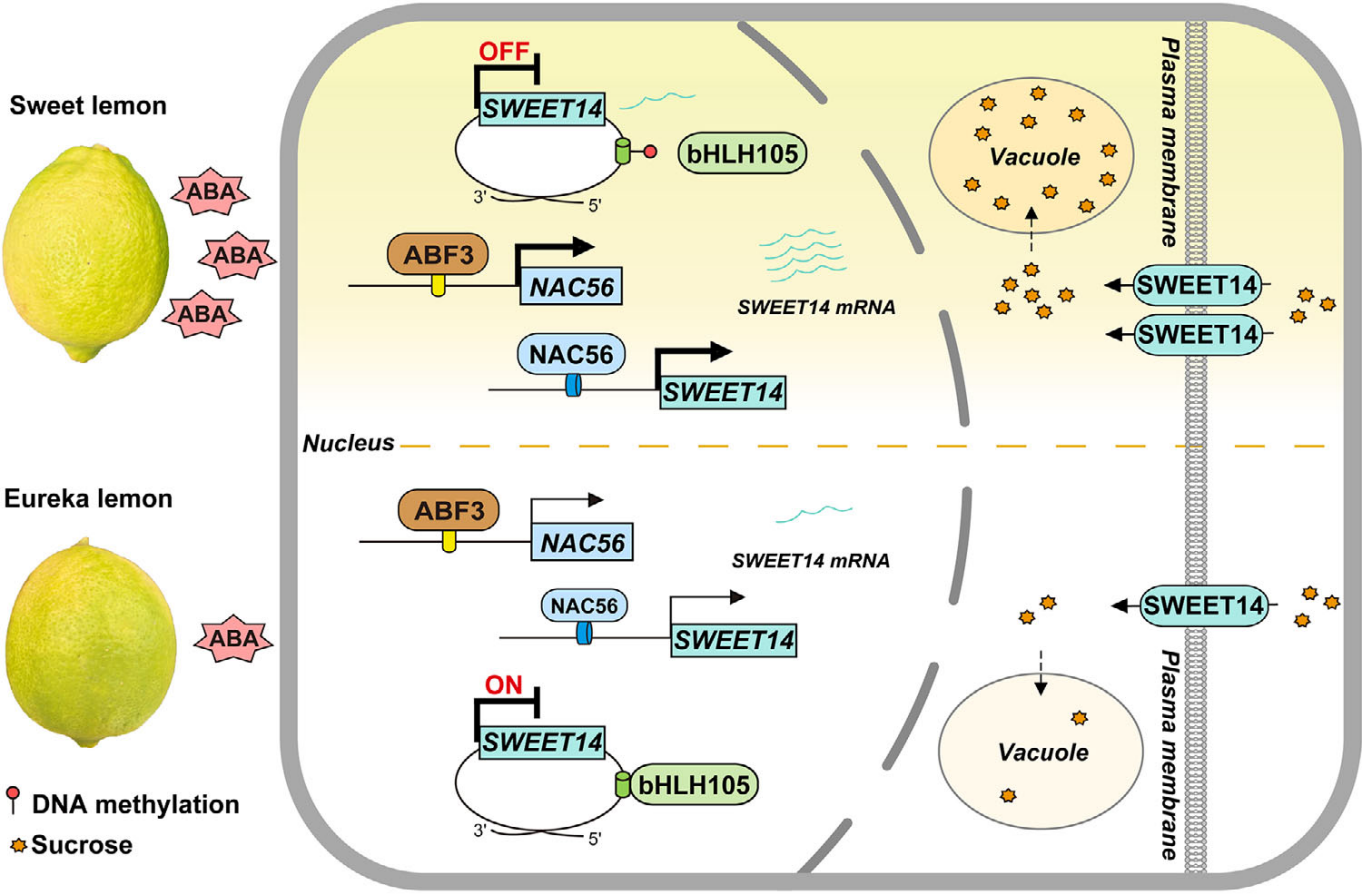
A proposed working model for illustrating the difference in *CliSWEET14* expression and sucrose accumulation between Eureka and Sweet lemon. The 3'‐UTRs of *CliSWEET14* from both lemons form a chromatin loop with their promoters, bringing the CACGAT motif into physical proximity with their intrinsic promoter regions. In Eureka lemon, ClibHLH105 binds to the unmethylated CACGAT motif within the 3'‐UTR and transcriptionally represses *CliSWEET14*. In contrast, hypermethylation of the second cytosine in the CACGAT motif in sweet lemon impairs ClibHLH105 binding, leading to relief of transcriptional repression on *CliSWEET14*. Additionally, the presence of higher ABA content in sweet lemon fruit relative to Eureka induces the ABA signaling pathway to a greater degree, which acts through the CliABF3*‐CliNAC56* regulatory cascade to upregulate *CliSWEET14* expression in a more powerful way. These two processes lead to stronger activation of *CliSWEET14* and consequently enhance sucrose transport, contributing to higher sucrose accumulation.

## 4 Materials and Methods

### 4.1 Plant Materials and Treatment

The EL fruits at 65, 100, 135, 175, and 205 DAF, leaves and flowers were collected from the Citrus Germplasm Repository of Huazhong Agricultural University (Wuhan, China). The SL fruits were collected at the same time from Guizhou province. Three biological replicates composed of six fruits with uniform size and ripening stage were sampled for each developmental stage. Embryogenic calli of Satsuma mandarin (*C. unshiu*) on solid MT basal medium containing 40 g/L sucrose and 0.7% agar (pH 5.8) was maintained in a culture room under a 16‐h light/8‐h dark photoperiod at 25°C. 5‐Aza treatment was carried out according to the previous method [63] with slight modifications. To this purpose, 0.3 mL of 5‐Aza (Sigma) solution (36 mm) was injected into the pulp of SL fruits, using an equal volume of water injected into the opposite side as a mock control. Injections were performed on a total of 15 fruit, randomly divided into three biological replicates, for five times at 1‐day intervals. Fruit pulps were harvested 1 day after the last injection. All samples were frozen in liquid nitrogen, then stored at −80°C for further analysis.

### 4.2 Genome Sequencing and Assembly

High‐molecular‐weight DNA was extracted from SL fruits flesh. Libraries were constructed for PacBio HiFi, ultra‐long ONT reads, and Illumina sequencing, and sequenced on the Sequel II, PromethION and DNBSEQ‐T7 at the Genome Center of onemore tech (Wuhan, China), respectively. HiFi and ONT reads were independently assembled into contigs using HiFiasm and NextDenovo. The HiFiasm assembly was scaffolded into chromosome‐scale pseudomolecules using 3d‐DNA guided by Hi‐C data, followed by manual curation with Juicebox [64]. Assembly gaps were closed through two iterative rounds of polishing with HiFi reads and NextDenovo contigs. To distinguish the paternal and maternal haplotypes, we leveraged the known progenitors, sour orange (SO) and citron (CIT), of lemon, and those of SO originating from the hybridization of mandarin and pummelo. We separately mapped the resequencing data of mandarin, pummelo, and citron to the 18 pseudochromosomes. SNP density was calculated within sliding windows (window size: 1 kb step), and the origin of each window was assigned to mandarin, pummelo, or citron according to its SNP‐density profile. The haplotype origin of each chromosome was then determined as either citron‐derived (Haplotype A, CIT) or sour‐orange‐derived (Haplotype B, SO).

### 4.3 Genome Annotation

Repetitive sequences and transposable elements were identified and soft‐masked using RepeatMasker Tarailo‐Graovac and Chen [65]. Protein‐coding genes were predicted through an integrative strategy combining ab initio modeling, homology‐based inference, and RNA‐seq evidence. Ab initio predictions were generated with GlimmerHMM [66] and SNAP [67]. Homology‐based predictions were derived by aligning proteins from the CPBD (http://citrus.hzau.edu.cn/) against the masked assembly using GenomeThreader [68]. Transcript support was provided by *de novo* and genome‐guided assemblies constructed with Trinity (based on previously published RNA‐seq data [7] and integrated via the PASA pipeline [69, 70]. All evidence sources were consolidated with EVidenceModeler [70], followed by PASA‐mediated refinement to annotate UTRs and alternative splice variants [69]. Functional annotation was performed by sequence similarity searches against UniProt (http://www.uniprot.org/) using Diamond [72], and assignment of Gene Ontology and KEGG terms using eggNOG‐Mapper [73].

### 4.4 Gas Chromatography

Sugar levels were determined by gas chromatography (GC) with minor modifications based on a previously established protocol [45]. Soluble sugars were extracted from plant samples with 75% (v/v) methanol, and ribitol was added as an internal standard for quantitative normalization. Sugar extracts were derivatized first with methoxyamine hydrochloride and subsequently with *N*‐methyl‐*N*‐trimethylsilyl‐trifluoroacetamide. A GC9720Plus instrument (Fuli Instruments, Zhejiang, China) was used for sugar detection, and data analysis was performed via the internal standard method (FL97Plus.lnk). Each sample was tested in three technical replicates to guarantee experimental reproducibility.

### 4.5 RNA Extraction and qPCR Analysis

Total RNA was isolated from frozen fruits and calli tissues using TRIzol reagent (Invitrogen, Carlsbad, CA, USA), followed by DNase I treatment to eliminate genomic DNA contamination. First‐strand cDNA was synthesized from 1 µg of total RNA using HiScript II Q RT SuperMix (Vazyme, Nanjing, China). Quantitative PCR (qPCR) was performed using ChamQ Universal SYBR qPCR Master Mix (Cat. Q771; Vazyme, Nanjing, China) on the Step One Plus Real‐Time PCR System (Applied Biosystems, Thermo Fisher Scientific Corp., USA). The qPCR protocol consisted of an initial denaturation at 95°C for 30 s, followed by 45 cycles of 95°C for 15 s and 60°C for 30 s. Melting curve analysis was performed to verify amplification specificity. Quantification of gene expression was achieved using the 2^−ΔΔCt^ method [3], with *CliACTIN* as internal reference gene. Each experiment was conducted in triplicate. All primers are listed in Table S5.

### 4.6 Subcellular Localization Analysis

The coding sequences (CDSs) of *CliSWEET14*, *CliNAC56*, and *CliABF3* (without stop codons) were amplified by PCR and cloned in‐frame with the yellow fluorescent protein (YFP) gene into the p101LYFP vector, which is driven by the CaMV 35S promoter, to generate YFP fusion plasmids. These recombinant constructs, together with the empty p101LYFP vector (negative control), were transformed into *Agrobacterium tumefaciens* strain GV3101 separately. Agrobacterial cultures were incubated overnight in LB medium, collected by centrifugation, and resuspended in infiltration buffer (10 mM MgCl_2_, 10 mm MES, pH 5.7). mCherry‐tagged VirD2NLS was utilized as a nuclear marker to verify nuclear localization. Four‐week‐old *N. benthamiana* leaves were co‐infiltrated with agrobacterial suspensions adjusted to an OD_600_ of 0.4. Fluorescence signals were observed 36 to 48 h after infiltration using a Leica TCS‐SP8 confocal laser‐scanning microscope (Leica Microsystems, Wetzlar, Germany). YFP fluorescence was excited at 514 nm, and emitted light was collected within the 530–560 nm wavelength range.

### 4.7 In Situ Hybridization

Juice sacs from *SL* fruits at 135 days after full blossom (DAFB) were fixed and processed for in situ hybridization to determine the spatial expression pattern of *CliSWEET14*. The RNASweAMI Plant in situ Hybridization Kit (BCIP/nitroblue tetrazolium (NBT) detection; Cat. GF011; Servicebio, Wuhan, China) was employed following the manufacturer's standard procedure to visualize *CliSWEET14* transcript localization in juice sac tissues.

### 4.8 ^14^C‐Suc Uptake Assay

^14^C‐Suc uptake assay was carried out according to the previous description with slight changes [74]. Yeast strain CSY4000 carrying either pDR196‐*CliSWEET14* or the empty pDR196 vector (control) was cultured in liquid minimal medium supplemented with maltose at 30°C until reaching an optical density at 623 nm (OD_623_) of approximately 0.8. Yeast cells were harvested by centrifugation, washed twice with 25 mm sodium phosphate buffer (pH 5.0), and resuspended in the same buffer to an OD_623_ of 20. Uptake reactions were initiated by adding ^14^C‐Suc (0.05 µCi) to the yeast suspension to a final concentration of 20, 30, 50, 125, 200, and 250 µm, followed by incubation in a 30°C shaking incubator. Samples were collected at predetermined time points, centrifuged at 4°C, rinsed twice with distilled water, and resuspended in 200 µL of distilled water. The resuspended cells were transferred to scintillation tubes containing liquid scintillation cocktail, and radioactivity was measured using a Tri‐Carb 2810TR multifunctional scintillation counter (PerkinElmer, Tri‐Carb 2810TR, Singapore).

### 4.9 PCR Amplification with Methylation‐Sensitive Restriction Enzyme (Chop‐PCR)

Genomic DNA (gDNA) was isolated from plant tissues using the cetyltrimethylammonium bromide (CTAB) method. A 500‐ng portion of purified gDNA was digested overnight at 37°C with the methylation‐sensitive restriction enzyme AciI (New England Biolabs, catalog no. R0551V). The digested gDNA was then used as a template for PCR amplification to evaluate methylation status. Primers used for Chop–PCR are provided in Table S5.

### 4.10 Bisulfite Sequencing‐based DNA Methylation Analysis

Bisulfite conversion of gDNA was carried out using the EZ DNA Methylation‐Gold Kit (Zymo Research, catalog no. D5005) according to the manufacturer's protocol. Target genomic regions were amplified by PCR from bisulfite‐converted DNA using specific primers (Table S5). PCR products were purified, cloned into the pEASY‐Blunt vector (TransGen Biotech, catalog no. CB101‐01), and sequenced via Sanger sequencing. Sequencing reads were analyzed with Kismeth software (version 74) to calculate the percentage of methylated cytosines. For amplicon‐based deep sequencing of DNA methylation, bisulfite‐converted gDNA was first amplified with target‐specific primers. A second PCR round was performed to attach barcodes to both ends of the amplicons for library construction. Equimolar amounts of barcoded PCR products were pooled and sequenced on the NovaSeq 6000 platform (Annoroad Gene Technology). Sequencing data were processed and analyzed using HiMeth software (http://www.hi‐tom.net/Hi‐TOM_meth/).

### 4.11 Chromosome Conformation Capture (3C) Assay

For each 3C replicate, approximately 5.0 g of pulp from SL or EL was cross‐linked in nuclei isolation buffer containing 1% formaldehyde at 4°C for 10 min. Cross‐linking was terminated by adding 0.15 m glycine and incubating at 4°C for 5 min. Subsequent procedures followed the maize 3C protocol [32, 75], except the digestion with 50 U *Dpn*II and ligation with 20 U T4 DNA ligase. A genomic fragment lacking *Dpn*II restriction sites served as negative control. All 3C primer sequences are provided in Table S5.

### 4.12 ABA Content Determination

Endogenous ABA levels were determined following Sun [76]. Separation was achieved using an Agilent 1100 HPLC system coupled with an Agilent API3000 triple‐quadrupole mass spectrometer. Deuterated ABA (^2^H_6_‐ABA) was utilized as internal standard. A minimum of three biological replicates were analyzed for each sample.

### 4.13 Histochemical Assay of GUS Activity

Promoter regions of *CliSWEET14* (1,422 bp upstream of the translation start site) and *CliNAC56* (602 bp upstream) were amplified from genomic DNA and cloned into the DX2181G vector upstream of the *GUS* coding sequence, generating pSWEET14:GUS and pNAC56:GUS, respectively. Satsuma mandarin calli were transformed with *Agrobacterium* strains harboring reporter constructs or empty vector and incubated at 25°C in darkness for 2 days. GUS activity was detected using a commercial staining kit (Coolaber, Beijing, China) according to the manufacturer's instructions.

### 4.14 Transient Infiltration of Juice Sacs

Full‐length coding sequences of *CliSWEET14*, *ClibHLH105*, *CliNAC56*, and *CliABF3* were cloned into pENTR1A using the ClonExpress II One‐Step Cloning Kit (Vazyme, Nanjing, China) and subsequently transferred via LR Gateway (Thermo Fisher) into pK7WG2D (35S‐driven) for overexpression. The resulting constructs, together with empty vector controls, were introduced into *Agrobacterium tumefaciens* EHA105 (Weidibio, Shanghai, China). Bacterial cultures were grown to OD_600_ = 0.8 at 28°C, harvested by centrifugation (5500 rpm), and resuspended in infiltration buffer [10 mm MES, 10 mm MgCl_2_, 50 µm acetosyringone (pH 5.7)]. Juice sacs were vacuum‐infiltrated with *Agrobacterium* suspensions for 45 min. Primers are listed in Table S5.

### 4.15 Transformation of Citrus Calli

Overexpression constructs were identical to those used for juice sac infiltration. For RNA interference (RNAi), 250‐bp gene‐specific fragments of *CliSWEET14*, *CliNAC56*, and *CliABF3* were inserted into pDONR221 and subsequently recombined into the 35S‐driven pGWB‐RNAi vector (Thermo Fisher) via Gateway cloning. Both overexpression and RNAi constructs were transformed into *A. tumefaciens* EHA105 (Weidibio, Shanghai, China). Suspension‐cultured calli were blotted dry, immersed in MT liquid medium containing *Agrobacterium* EHA105 (OD_600_ = 0.8) harboring the target construct, and vacuum‐infiltrated as described [45]. Transformants were selected on antibiotic‐containing medium and verified by qPCR. Primers are provided in Table S5.

### 4.16 Yeast One‐Hybrid (Y1H) Assay

The promoter regions of *CliSWEET14* (1442 bp upstream of the translation start site) and *CliNAC56* (602 bp upstream of the translation start site) were PCR‐amplified from citrus genomic DNA and cloned into the pAbAi vector, which had been pre‐digested with *Kpn*I and *Xho*I. The resulting bait plasmids were linearized with *Bbs*I and integrated into the Y1HGold yeast strain. The CDSs of *ClibHLH105*, *CliNAC56*, and *CliABF3* were inserted into the pGADT7 vector between the *Nde*I and *EcoR*I restriction sites to generate prey constructs. All bait‐prey construct pairs, along with positive controls (pGAD‐p53 combined with p53‐AbAi) and negative controls (empty pGADT7‐AD vector co‐transformed with each bait plasmid), were co‐transformed into the Y1HGold yeast strain. Y1H assays were carried out in strict accordance with the instructions provided in the Yeast Transformation System 2 user manual (Clontech, Mountain View, CA, USA).

### 4.17 ChIP‐qPCR

According to the methods previously described [77], the full‐length CDS of *CliABF3* was fused to the pK7WG2D expression vector containing a FLAG epitope tag, following a previously described method [77]. The 35S:CliABF3‐FLAG construct and the empty 35S: FLAG vector (control) were transformed into *Agrobacterium tumefaciens* strain GV3101 and transiently expressed in citrus calli. The citrus calli displaying green fluorescence were collected 10 days after infiltration for ChIP–qPCR analysis. Approximately 2 g of calli was fixed in 1% (w/v) formaldehyde, and the cross‐linking reaction was quenched by adding 125 mm glycine. Chromatin was sheared into 250–600 bp fragments using a Bioruptor plus Sonicator (Belgium) and immunoprecipitated with anti‐FLAG Magnetic Beads (GenScript, Cat. L00835). The purified immunoprecipitated DNA was resuspended in distilled water and quantified by qPCR using gene‐specific primers. The enrichment level of target DNA fragments was calculated as previously reported [78]. All data are presented as the mean ± standard deviation (SD) from three independent biological replicates. Primers used for ChIP‐qPCR are listed in Table S5.

### 4.18 Electrophoretic Mobility Shift Assay (EMSA)

The CDSs of *CliNAC56* and *CliABF3* were cloned into the pHMGWA vector and transformed into *Escherichia coli* Rosetta (DE3) competent cells (Weidi Biotechnology, Shanghai, China). The CliNAC56‐His and CliABF3‐His fusion proteins were induced with 0.45 mm isopropyl β‐D‐1‐thiogalactopyranoside (IPTG) at 16°C for 16 h and purified using Ni‐NTA Agarose (Qiagen, Germany). Biotin‐labeled DNA probes containing normal, mutated or methylated forms of the examined motifs were synthesized by Tsingke Biological Technology (Beijing, China), with unlabeled probes serving as competitors. Protein‐DNA binding interactions were detected using an EMSA kit (Beyotime, Shanghai, China), and chemiluminescent signals were captured with a Tanon 5200 imaging system (Shanghai, China). Sequences of all probes used are provided in Table S5.

### 4.19 Dual LUC Assay

For reporter constructs, the promoter sequences of *CliSWEET14* and *CliNAC56* were cloned into the pGreen II 0800‐Luc reporter vector [79]. For effector constructs, the full‐length CDSs of *ClibHLH105*, *CliNAC56*, and *CliABF3* were inserted into different overexpression vectors carrying distinct epitope tags. Effector and reporter constructs were co‐infiltrated into the leaves of *N. benthamiana* plants. Infiltrated plants were maintained under long‐day conditions (16‐h light/8‐h dark) at 23°C for 48–72 h before analysis. For luciferase activity detection, infiltrated leaf tissues were either harvested for direct quantification or sprayed with 0.1 mm D‐luciferin potassium salt (Coolaber, Beijing, China). LUC activity was measured using the Dual‐Luciferase Reporter Assay Kit (Cat. E1910; Promega, Madison, WI, USA). Luminescence signals were detected using either an Infinite 200 Pro microplate reader (Tecan, Mannedorf, Switzerland) or a NightSHADE LB 985 imaging system (Berthold, Germany). The assay was performed with three independent biological replicates for each treatment.

### 4.20 Statistical Analysis

Data are presented as mean ± SD (standard deviation) of three biological replicates. No data transformation, normalization, or outlier exclusion was applied, except for ^14^C‐sucrose uptake kinetics, which were analyzed using the Lineweaver–Burk to estimate Km. Pairwise comparisons were performed using two‐tailed Student's *t*‐tests. Each treatment was compared only with its corresponding control, and no multiple comparison correction was applied because the comparisons were pre‐planned and limited. All analyses were conducted using GraphPad Prism v10.0, with significance set at ^*^ *p* < 0.05, ^**^ *p* < 0.01, and ^***^ *p* < 0.001.

### 4.21 Accession Numbers

Genome assemblies have been deposited in the NGDC BioProject (https://www.cncb.ac.cn/) under accession no. PRJCA043764. Gene IDs from this study can be found in the uploaded genomic data: *CliSWEET14* (Csli_CIT_5g_031560), *ClibHLH105* (Csli_SO_2g_010310), *CliNAC56* (Csli_CIT_4g_011970), and *CliABF3* (Csli_CIT_8g_004450).

## Author Contributions

J.H.L., C.L.L., Y.K.Z. and Y W planned and designed the research. Y.K.Z. and Y W performed the experiments and analyzed the data, with the assistance of Y.L.W. and H.W.C. completed genome assembly and bioinformatics data analysis, and Q.Y.C., W.X., A.Q.Z., X.J., X.M.S., Z.C.Y., X.Z., and W.Q.H. assisted with bioinformatics analysis. Y.K.Z. and Y.W. wrote the manuscript draft. C.L.L. and J.H.L. finalized writing and revision of the manuscript. All authors have read and approved the final version of the manuscript.

## Conflicts of Interest

The authors declare no conflicts of interest.

## Supporting information




**Supporting File 1**: advs78518‐sup‐0001‐SuppMat.docx.

**Supporting File 2**: advs78518‐sup‐0002‐TableS1‐S5.docx.

## Data Availability

The data that support the findings of this study are available on request from the corresponding author. The data are not publicly available due to privacy or ethical restrictions.
