# Supplementary material for: 3′‐UTR Hypermethylation and ABA Signaling Coordinate Transcriptional Activation of SWEET14 for High Sugar Accumulation in Citrus
Source: Adv Sci (Weinh). 2026 Oct 31:e78518. Online ahead of print. doi: 10.1002/advs.78518 (PMC13723718; doi:10.1002/advs.78518)
Supplement: Supplementary file 1 [file ADVS-9999-e78518-s001.docx]

**Supplementary figures**

**Supplementary Figure S1.** Genome survey of Sweet lemon.

**Supplementary Figure S2**. Soluble sugar accumulation and functional enrichment of co-expressed genes in the sugar metabolism module.

**Supplementary Figure S3.** Venn diagram illustrating overlaps among gene sets significantly correlated with fructose, glucose and sucrose.

**Supplementary Figure S4**. *CliSWEET14* and *CliERD6L7* expression level revealed by RNA-seq.

**Supplementary Figure S5**. The expression levels of *CliSWEET14* and sugar contents in transgenic citrus juice sacs with the overexpressing *CliSWEET14*.

**Supplementary Figure S6**. The expression levels of *ClibHLH105* and *ClibHLH13* revealed by RNA-seq.

**Supplementary Figure S7**. DNA methylation variation of *CliSWEET14* was confirmed by bisulfite sequencing in SL juice sacs following transient overexpression of *ClibHLH105*, both in the presence and absence of 5-Aza treatment.

**Supplementary Figure S8**. Identification of putative *cis*-regulatory elements within the *CliSWEET14* promoter region.

**Supplementary Figure S9**. The expression level of *CliNAC56* in the leaves, stems, flowers, peels, pulps, and roots of Sweet lemon.

**Supplementary Figure S10**. The expression level of *CliNAC56* and *CliSWEET14* in Ri-EV calli with ABA treatment.

**Supplementary Figure S11.** Putative ABRE motifs in the *CliNAC56* promoter.

**Supplementary Figure S12.** The transcript level *CliABFs* revealed by RNA-seq.

**Supplementary Figure S13.** Cellular localization and transcriptional activation activity of CliABF3.

**Supplementary Figure S1.** Genome survey of Sweet lemon. Kmer size = 17.

**Supplementary Figure S2**. Soluble sugar accumulation and functional enrichment of co-expressed genes in the sugar metabolism module.

**A)** Comparison of DEG expression patterns with the developmental accumulation profiles of sucrose, fructose, and glucose. The sugar-associated groups describe correlations with measured sugar levels and do not imply sugar-specific responsiveness.

**B)** GO enrichment analysis of co-expressed genes soluble sugars module.

**Supplementary Figure S3.** Venn diagram illustrating overlaps among gene sets significantly correlated with fructose, glucose and sucrose. The bar chart shows total gene counts for each sugar correlated group.

**Supplementary Figure S4.** The expression level of *CliSWEET14* and *CliERD6L7* revealed by RNA-seq.

**A)** The FPKM value of *CliSWEET14*.

**B)** The FPKM value of *CliERD6L7*.

Error bars indicate ± SD (*n* = 3). Two-tailed Student’s t-test was conducted for analyzing the significant difference (**P* < 0.05, ***P* < 0.01, ****P* < 0.001).

**Supplementary Figure S5**. The expression levels of *CliSWEET14* and sugar contents in transgenic citrus juice sacs with the overexpressing *CliSWEET14*.

**A)** The expression of *CliSWEET14* in OE-*CliSWEET14* and OE-EV samples.

**B)** The sugar contents in OE-*CliSWEET14* and OE-EV samples.

Error bars indicate ± SD (*n* = 3). Two-tailed Student’s t-test was conducted for analyzing the significant difference (**P* < 0.05, ****P* < 0.001). FW, fresh weight.

**Supplementary Figure S6**. The expression level of *ClibHLH105* and *ClibHLH13* revealed by RNA-seq.

**A)** The FPKM value of *ClibHLH105*.

**B)** The FPKM value of *ClibHLH13*.

Error bars indicate ± SD (*n* = 3). Two-tailed Student’s t-test was conducted for analyzing the significant difference (****P* < 0.001).

**Supplementary Figure S7**. DNA methylation variation of *CliSWEET14* was confirmed by bisulfite sequencing in Sweet lemon juice sacs following transient overexpression of *ClibHLH105*, both in the presence and absence of 5-Aza treatment.

**Supplementary Figure S8**. Identification of putative *cis*-regulatory elements within the *CliSWEET14* promoter region.

**Supplementary Figure S9**. The expression level of *CliNAC56* in the leaves, stems, flowers, peels, pulps, and roots of Sweet lemon.

Error bars indicate ± SD (*n* = 3). Two-tailed Student’s t-test was conducted for analyzing the significant difference (****P* < 0.001).

**Supplementary Figure S10.** The expression level of *CliNAC56* and *CliSWEET14* in Ri-EV calli with ABA treatment.

**A)** The expression of *CliNAC56*.

**B)** The expression of *CliSWEET14*.

Error bars indicate ± SD (*n* = 3). Two-tailed Student’s t-test was conducted for analyzing the significant difference (****P* < 0.001).

**Supplementary Figure S11.** Putative ABRE motifs in the *CliNAC56* promoter.

**Supplementary Figure S12.** The transcript level *CliABFs* revealed by RNA-seq. Error bars indicate ± SD (*n* = 3).

**Supplementary Figure S13.** Cellular localization and transcriptional activation activity of CliABF3.

**A)** The subcellular localization of CliABF3-YFP in leaf epidermal cells of *N. benthamiana*. mCherry (mCherry-VirD2NLS) works as a nucleus marker. Scales bar=20 μm.

**B)** Growth of yeast cells strain AH109 transformed with different vectors, using BD (pGBKT7) as a negative control, on Synthetic Dropout (SD)/-T (Trp), and SD/-T (Trp)/-H (His) /-A (Ade) added with X-α-gal.
