# Supplementary material for: 3′‐UTR Hypermethylation and ABA Signaling Coordinate Transcriptional Activation of SWEET14 for High Sugar Accumulation in Citrus
Source: Adv Sci (Weinh). 2026 Oct 31:e78518. Online ahead of print. doi: 10.1002/advs.78518 (PMC13723718; doi:10.1002/advs.78518)
Supplement: Supplementary file 2 [file ADVS-9999-e78518-s002.docx]

**Supplementary Tables**

**Supplementary Table S1.** Summary of the sequencing data for Sweet lemon.

**Supplementary Table S2.** The list of co-expressed genes in soluble sugar modules.

**Supplementary Table S3.** Summary of bisulfite sequencing and alignment data for each sample.

**Supplementary Table S4.** Potential *CliSWEET14* regulating proteins identified by Y1H screening.

**Supplementary Table S5.** List of primers or EMSA probes used in this study.

| **Sequence Type** | **Raw data (Gb)** | **Tissue** | **Alignment Rate** |
| --- | --- | --- | --- |
| HiFi | 26.13 | flesh | \ |
| illumina | 35.28 | flesh | 99.52% |
| Hi-C | 37.19 | flesh | \ |
| ONT Ultra-long | 38.99 | flesh | \ |

**Supplementary Table S1. Summary of the sequencing data for Sweet lemon.**

**Supplementary Table S2. Co-expressed genes in soluble sugar modules**

| **Gene ID** | **Function Description** |
| --- | --- |
| Csli_CIT_5g_019700 | 2-oxoglutarate dehydrogenase |
| Csli_CIT_7g_021930 | receptor-like serine threonine-protein kinase |
| Csli_CIT_9g_016220 | E3 ubiquitin protein ligase |
| Csli_CIT_7g_007340 | transcription factor |
| Csli_CIT_5g_001490 | hypothetical protein WN943_013057 |
| Csli_CIT_3g_005180 | E3 ubiquitin-protein ligase |
| Csli_CIT_7g_000430 | Belongs to the glycosyl hydrolase 1 family |
| Csli_CIT_1g_008060 | Belongs to the cytochrome P450 family |
| Csli_CIT_9g_020720 | Vacuolar protein-sorting-associated protein 37 homolog |
| Csli_CIT_2g_013700 | aarF domain-containing protein kinase At1g79600 |
| Csli_CIT_3g_010880 | Belongs to the heat shock protein 70 family |
| Csli_CIT_5g_031130 | Sugar transporter (TC 2.A.1.1) family ERD6L7 |
| Csli_CIT_7g_021940 | receptor-like serine threonine-protein kinase |
| Csli_CIT_1g_014540 | ADP binding |
| Csli_CIT_4g_014610 | acyl-activating enzyme |
| Csli_CIT_5g_026940 | ER lumen |
| Csli_CIT_6g_024120 | germin-like protein |
| Csli_CIT_3g_014360 | belongs to the protein kinase superfamily |
| Csli_CIT_8g_002970 | Cysteine-rich and transmembrane domain-containing protein |
| Csli_CIT_6g_003050 | Protein GLUTAMINE DUMPER |
| Csli_CIT_8g_017170 | Uncharacterized protein |
| Csli_CIT_4g_005810 | CAAX amino terminal protease family protein |
| Csli_CIT_5g_006130 | 4-coumarate--CoA ligase-like |
| Csli_CIT_7g_023600 | Telomere repeat-binding protein |
| Csli_CIT_8g_007210 | Essential component of the vacuolar proton pump (V- ATPase) |
| Csli_CIT_5g_025610 | germin-like protein |
| Csli_CIT_7g_022210 | Uncharacterized protein |
| Csli_CIT_1g_005690 | Belongs to the glycosyl hydrolase 9 (cellulase E) family |
| Csli_CIT_1g_014530 | ADP binding |
| Csli_CIT_5g_007610 | Belongs to the acyl-CoA oxidase family |
| Csli_CIT_8g_001450 | Uncharacterized protein |
| Csli_CIT_4g_009710 | Plant mobile domain |
| Csli_CIT_2g_024000 | G-type lectin S-receptor-like serine threonine-protein kinase |
| Csli_CIT_5g_020170 | hypothetical protein CUMW_233650 |
| Csli_CIT_9g_003800 | Alpha beta-Hydrolases superfamily protein |
| Csli_CIT_7g_018530 | proton transmembrane transport |
| Csli_CIT_7g_022260 | Belongs to the iron ascorbate-dependent oxidoreductase family |
| Csli_CIT_3g_015250 | Fantastic Four meristem regulator |
| Csli_CIT_6g_010270 | BEST Arabidopsis thaliana protein match is |
| Csli_CIT_5g_014970 | Belongs to the cytochrome P450 family |
| Csli_CIT_7g_003670 | exocyst complex component |
| Csli_CIT_2g_023890 | synthase |
| Csli_CIT_2g_029390 | Uncharacterized protein |
| Csli_CIT_8g_017180 | Uncharacterized protein |
| Csli_CIT_5g_006120 | Catalyzes the reduction of fatty acyl-CoA to fatty alcohols |
| Csli_CIT_4g_008630 | Dynamin Fzo YdjA family |
| Csli_CIT_2g_028720 | phosphatidate cytidylyltransferase 1 isoform X2 |
| Csli_CIT_5g_005170 | U-box domain-containing protein |
| Csli_CIT_5g_037730 | Beach domain-containing protein |
| Csli_CIT_6g_021580 | Belongs to the MIP aquaporin (TC 1.A.8) family |
| Csli_CIT_4g_006800 | Peptidase inhibitor I9 |
| Csli_CIT_5g_013480 | Protein SHOOT GRAVITROPISM |
| Csli_CIT_2g_023540 | isoform X1 |
| Csli_CIT_4g_004400 | Calmodulin-binding receptor-like cytoplasmic kinase |
| Csli_CIT_3g_010940 | REF SRPP-like protein |
| Csli_CIT_2g_027830 | Belongs to the cullin family |
| Csli_CIT_3g_023230 | nucleoredoxin 2 |
| Csli_CIT_5g_032840 | Uncharacterized protein |
| Csli_CIT_3g_017770 | dof zinc finger protein |
| Csli_CIT_4g_006100 | Inter-alpha-trypsin inhibitor heavy |
| Csli_CIT_4g_018650 | ethanolamine kinase |
| Csli_CIT_4g_016990 | E3 ubiquitin-protein ligase |
| Csli_CIT_7g_020660 | Autophagy-related protein |
| Csli_CIT_3g_018480 | Tryptophan aminotransferase-related protein |
| Csli_CIT_5g_005510 | cytochrome P450 |
| Csli_CIT_9g_019660 | Pentatricopeptide repeat-containing protein At3g49170 |
| Csli_CIT_2g_000540 | Importin-5-like |
| Csli_CIT_1g_005480 | Condensin-2 complex subunit |
| Csli_CIT_5g_012210 | Partial alpha/beta-hydrolase lipase region |
| Csli_CIT_2g_026140 | IQ-domain |
| Csli_CIT_3g_015470 | Auxin response factors (ARFs) |
| Csli_CIT_7g_017730 | exocyst complex component |
| Csli_CIT_7g_013860 | NAC domain-containing protein |
| Csli_CIT_6g_001020 | Galectin |
| Csli_CIT_8g_017590 | RING-H2 finger protein |
| Csli_CIT_3g_018730 | Shikimate kinase |
| Csli_CIT_6g_014440 | Lectin-domain containing receptor kinase |
| Csli_CIT_4g_017000 | PP2C and cyclic nucleotide-binding kinase domain-containing |
| Csli_CIT_3g_017140 | Target of Myb protein |
| Csli_CIT_5g_012040 | Uncharacterized protein |
| Csli_CIT_3g_007930 | Belongs to the iron ascorbate-dependent oxidoreductase family |
| Csli_CIT_6g_002320 | AP-4 complex subunit |
| Csli_CIT_5g_033460 | Ethylbenzene dehydrogenase |
| Csli_CIT_5g_034510 | UNC93-like protein |
| Csli_CIT_5g_001660 | Uncharacterized protein |
| Csli_CIT_1g_014070 | Ribonuclease 3-like protein |
| Csli_CIT_8g_017690 | Transcription initiation factor TFIID subunit |
| Csli_CIT_5g_031740 | Belongs to the cullin family |
| Csli_CIT_1g_014390 | Belongs to the disease resistance NB-LRR family |
| Csli_CIT_2g_023110 | Protein FAR1-RELATED SEQUENCE |
| Csli_CIT_1g_013390 | LRR receptor-like serine threonine-protein kinase |
| Csli_CIT_5g_002250 | Belongs to the sterol desaturase family |
| Csli_CIT_8g_021450 | cytochrome P450 |
| Csli_CIT_7g_018180 | Synaptotagmin-3-like |
| Csli_CIT_9g_012860 | Cytokinin dehydrogenase |
| Csli_CIT_5g_011010 | exocyst complex component |
| Csli_CIT_8g_025840 | Lectin-domain containing receptor kinase |
| Csli_CIT_8g_017520 | Belongs to the UDP-glycosyltransferase family |
| Csli_CIT_3g_021250 | Protein kinase superfamily protein |
| Csli_CIT_3g_020240 | Protein WEAK CHLOROPLAST MOVEMENT UNDER BLUE |
| Csli_CIT_7g_022200 | Uncharacterized protein |
| Csli_CIT_6g_014800 | phosphatase 2C |
| Csli_CIT_5g_025210 | Cinnamoyl-CoA reductase |
| Csli_CIT_6g_009150 | phosphatase |
| Csli_CIT_4g_009720 | Plant mobile domain |
| Csli_CIT_7g_005640 | Belongs to the actin family |
| Csli_CIT_4g_007540 | E3 ubiquitin-protein ligase |
| Csli_CIT_2g_010200 | kelch repeat-containing protein |
| Csli_CIT_4g_006520 | zinc finger |
| Csli_CIT_7g_012410 | FHA domain-containing protein |
| Csli_CIT_7g_003430 | Protein NRT1 PTR FAMILY |
| Csli_CIT_2g_027850 | F-box kelch-repeat protein |
| Csli_CIT_4g_023560 | Uncharacterized protein |
| Csli_CIT_7g_010360 | isoform X1 |
| Csli_CIT_9g_007550 | Protein LIGHT-DEPENDENT SHORT HYPOCOTYLS |
| Csli_CIT_4g_015830 | inactive protein kinase |
| Csli_CIT_7g_012330 | Metacaspase-1-like |
| Csli_CIT_4g_013030 | Ring finger protein |
| Csli_CIT_7g_001270 | transport) protein |
| Csli_CIT_1g_000620 | Endochitinase |
| Csli_CIT_7g_012100 | Polygalacturonase |
| Csli_CIT_5g_010670 | Belongs to the UDP-glycosyltransferase family |
| Csli_CIT_1g_006450 | kinase interacting family protein |
| Csli_CIT_2g_009650 | Nodulin-like |
| Csli_CIT_5g_034130 | Uncharacterized protein |
| Csli_CIT_3g_008680 | importin subunit |
| Csli_CIT_5g_004130 | calmodulin binding protein |
| Csli_CIT_6g_001850 | Belongs to the histidine acid phosphatase family |
| Csli_CIT_5g_025530 | Pentatricopeptide repeat-containing protein |
| Csli_CIT_4g_000980 | Belongs to the nonaspanin (TM9SF) (TC 9.A.2) family |
| Csli_CIT_3g_012760 | ATP coupled with the transport of calcium |
| Csli_CIT_5g_039000 | electron transfer activity |
| Csli_CIT_4g_007320 | Belongs to the glycosyltransferase 2 family |
| Csli_CIT_9g_003480 | Uncharacterized protein |
| Csli_CIT_6g_005630 | cytochrome P450 |
| Csli_CIT_3g_004180 | Disease resistance protein |
| Csli_CIT_1g_006460 | Uncharacterized protein |
| Csli_CIT_1g_009750 | alanine aminotransferase |
| Csli_CIT_3g_003620 | T-complex protein |
| Csli_CIT_5g_011510 | transcription factor |
| Csli_CIT_3g_007960 | Belongs to the iron ascorbate-dependent oxidoreductase family |
| Csli_CIT_9g_000640 | Vacuolar protein sorting-associated protein |
| Csli_CIT_1g_021600 | Belongs to the acyl-CoA oxidase family |
| Csli_CIT_1g_017560 | Glucose-inhibited division family A protein |
| Csli_CIT_4g_009100 | Belongs to the glycosyltransferase 31 family |
| Csli_CIT_9g_005620 | Heat Stress Transcription Factor |
| Csli_CIT_7g_012940 | transcription factor |
| Csli_CIT_7g_004950 | Alpha-glucan water dikinase |
| Csli_CIT_7g_003840 | UPF0392 protein |
| Csli_CIT_5g_007880 | Protein transport protein sec16 |
| Csli_CIT_4g_002860 | plasma membrane ATPase |
| Csli_CIT_6g_007030 | Belongs to the ABC transporter superfamily. |
| Csli_CIT_5g_003450 | Protein NRT1 PTR FAMILY |
| Csli_CIT_8g_026680 | BEST Arabidopsis thaliana protein match is |
| Csli_CIT_3g_007100 | Uncharacterized protein |
| Csli_CIT_7g_015540 | Belongs to the iron ascorbate-dependent oxidoreductase family |
| Csli_CIT_2g_024520 | sphingosine kinase |
| Csli_CIT_3g_005870 | isoform X1 |
| Csli_CIT_9g_002150 | phospholipase d |
| Csli_CIT_7g_006810 | Sphingosine-1-phosphate lyase |
| Csli_CIT_2g_012910 | Ubiquitin-like-conjugating enzyme |
| Csli_CIT_1g_017080 | Belongs to the protein kinase superfamily. |
| Csli_CIT_2g_005460 | Belongs to the protein kinase superfamily. |
| Csli_CIT_6g_022460 | Zinc finger CCCH domain-containing protein |
| Csli_CIT_5g_009640 | Plant family of unknown function (DUF810) |
| Csli_CIT_8g_022330 | Vacuolar protein sorting-associated protein 41 homolog |
| Csli_CIT_1g_007970 | ABC transporter C family member |
| Csli_CIT_3g_023740 | carboxypeptidase |
| Csli_CIT_6g_006560 | Uncharacterized protein |
| Csli_CIT_4g_010570 | Belongs to the small heat shock protein (HSP20) family |
| Csli_CIT_3g_005780 | Leucine Rich Repeat |
| Csli_CIT_5g_037710 | ntmc2t4,ntmc2type4 |
| Csli_CIT_1g_004060 | Converts zeaxanthin into antheraxanthin and subsequently violaxanthin |
| Csli_CIT_9g_004610 | Long chain acyl-CoA synthetase |
| Csli_CIT_1g_019010 | Serine threonine-protein kinase |
| Csli_CIT_1g_013920 | LRR receptor-like serine threonine-protein kinase |
| Csli_CIT_7g_001080 | Polyamine oxidase |
| Csli_CIT_7g_022120 | Clathrin is the major protein of the polyhedral coat of coated pits and vesicles |
| Csli_CIT_5g_024350 | Belongs to the cytochrome P450 family |
| Csli_CIT_1g_007530 | Belongs to the protein kinase superfamily |
| Csli_CIT_6g_005660 | cytochrome P450 |
| Csli_CIT_4g_021290 | phosphatase 2C |
| Csli_CIT_5g_022860 | Uncharacterized protein |
| Csli_CIT_8g_025830 | Lectin-domain containing receptor kinase |
| Csli_CIT_1g_018070 | Intron maturase, type II family protein |
| Csli_CIT_2g_008610 | Potassium transporter |
| Csli_CIT_6g_006840 | serine threonine-protein kinase |
| Csli_CIT_4g_010170 | cobra-like protein |
| Csli_CIT_2g_000160 | Uncharacterized protein |
| Csli_CIT_5g_001650 | Uncharacterized protein |
| Csli_CIT_9g_010730 | Potassium channel |
| Csli_CIT_1g_005470 | Condensin-2 complex subunit |
| Csli_CIT_9g_019840 | lob domain-containing protein |
| Csli_CIT_2g_013830 | hypothetical protein Patl1 |
| Csli_CIT_1g_006140 | Nodulation receptor kinase-like |
| Csli_CIT_1g_004130 | Uncharacterized protein |
| Csli_CIT_7g_009500 | Adaptor-related protein complex 5, beta 1 subunit |
| Csli_CIT_9g_013540 | Bis(5'-adenosyl)-triphosphatase-like |
| Csli_CIT_6g_000820 | (NAC) domain-containing protein |
| Csli_CIT_2g_015250 | Dihydrolipoyllysine-residue succinyltransferase component of 2-oxoglutarate dehydrogenase complex |
| Csli_CIT_3g_016830 | zinc finger |
| Csli_CIT_4g_004770 | N-terminal C2 in EEIG1 and EHBP1 proteins |
| Csli_CIT_7g_019520 | Caffeoylshikimate esterase-like |
| Csli_CIT_1g_018730 | PDDEXK-like family of unknown function |
| Csli_CIT_5g_034620 | calcium-binding protein |
| Csli_CIT_2g_024030 | serine threonine-protein kinase |
| Csli_CIT_8g_000010 | Belongs to the histone deacetylase family. HD Type 1 subfamily |
| Csli_CIT_6g_016170 | Transcription factor |
| Csli_CIT_2g_028120 | Agamous-like MADS-box protein |
| Csli_CIT_6g_002550 | Auxin transport protein |
| Csli_CIT_4g_011680 | F-box protein |
| Csli_CIT_1g_015680 | xylosyltransferase |
| Csli_CIT_5g_024450 | SANTA (SANT Associated) |
| Csli_CIT_9g_009880 | Non-lysosomal glucosylceramidase that catalyzes the conversion of glucosylceramide to free glucose and ceramide |
| Csli_CIT_8g_009420 | Monoacylglycerol lipase |
| Csli_CIT_4g_015880 | Belongs to the mitochondrial carrier (TC 2.A.29) family |
| Csli_CIT_9g_015650 | Small G protein family protein RhoGAP |
| Csli_CIT_5g_025500 | UDP-N-acetylglucosamine--peptide N-acetylglucosaminyltransferase SEC |
| Csli_CIT_2g_024760 | isoform X1 |
| Csli_CIT_5g_011890 | Uncharacterized protein |
| Csli_CIT_2g_021250 | Transmembrane protein 56-like |
| Csli_CIT_5g_031560 | Bidirectional sugar transporter SWEET14 |
| Csli_CIT_3g_001740 | Chloride channel protein |
| Csli_CIT_5g_024600 | Clathrin interactor EPSIN |
| Csli_CIT_6g_010350 | UDP-arabinose 4-epimerase |
| Csli_CIT_3g_023200 | atexp11,atexpa11,athexp alpha 1.14,exp11,expa11 |
| Csli_CIT_2g_006020 | Catalyzes the oxidation of L-aspartate to iminoaspartate |
| Csli_CIT_6g_003040 | Protein GLUTAMINE DUMPER |
| Csli_CIT_9g_012810 | Belongs to the glycosyl hydrolase 17 family |
| Csli_CIT_8g_026030 | Tropinone reductase homolog |
| Csli_CIT_3g_024390 | A reductase |
| Csli_CIT_5g_020790 | Belongs to the small heat shock protein (HSP20) family |
| Csli_CIT_6g_006500 | Beta-catenin-like protein |
| Csli_CIT_6g_022610 | Belongs to the peptidase A1 family |
| Csli_CIT_6g_007630 | Uncharacterized protein |
| Csli_CIT_2g_024010 | serine threonine-protein kinase |
| Csli_CIT_4g_019050 | Umecyanin-like |
| Csli_CIT_7g_007800 | Leucine rich repeat N-terminal domain |
| Csli_CIT_6g_010630 | vacuolar amino acid transporter |
| Csli_CIT_4g_016310 | May be involved in the degradation of misfolded endoplasmic reticulum (ER) luminal proteins |
| Csli_CIT_5g_004750 | anthranilate phosphoribosyltransferase |
| Csli_CIT_4g_004750 | at4g33100 |
| Csli_CIT_4g_005820 | SAUR family |
| Csli_CIT_1g_019960 | maturation of SSU-rRNA from tricistronic rRNA transcript (SSU-rRNA, 5.8S rRNA, LSU-rRNA) |
| Csli_CIT_7g_010700 | Uncharacterized protein |
| Csli_CIT_8g_002190 | Belongs to the STXBP unc-18 SEC1 family |
| Csli_CIT_5g_035480 | UV-stimulated scaffold protein A homolog |
| Csli_CIT_1g_018030 | Disease resistance protein |
| Csli_CIT_7g_018740 | syntaxin binding |
| Csli_CIT_8g_002540 | serine threonine-protein kinase |
| Csli_CIT_4g_012060 | COP1-interacting protein 7 |
| Csli_CIT_1g_018510 | shikimate |
| Csli_CIT_5g_030460 | Dynamin family |
| Csli_CIT_3g_003190 | ATP-dependent RNA helicase |
| Csli_CIT_7g_004760 | E3 ubiquitin-protein ligase |
| Csli_CIT_9g_019790 | Molybdenum cofactor sulfurase |
| Csli_CIT_5g_007100 | Belongs to the BI1 family |
| Csli_CIT_2g_000030 | Iron-sulfur cluster assembly protei |
| Csli_CIT_4g_006510 | zinc finger |
| Csli_CIT_1g_018040 | inositol-3-phosphate synthase activity |
| Csli_CIT_9g_003880 | mitogen-activated protein kinase |
| Csli_CIT_1g_017150 | actin binding |
| Csli_CIT_7g_000690 | Uncharacterized protein |
| Csli_CIT_6g_002130 | Adrenodoxin-like protein, mitochondrial |
| Csli_CIT_6g_013690 | copper transporter |
| Csli_CIT_1g_018060 | Intron maturase, type II family protein |
| Csli_CIT_3g_013200 | Belongs to the disease resistance NB-LRR family |
| Csli_CIT_5g_036000 | BAG family molecular chaperone regulator |
| Csli_CIT_5g_033240 | glutamyl endopeptidase, chloroplastic |
| Csli_CIT_3g_008940 | Protein MON2 homolog |
| Csli_CIT_2g_005280 | Non-specific serine threonine protein kinase |
| Csli_CIT_3g_006500 | Disease resistance protein |
| Csli_CIT_4g_016780 | malic enzyme |
| Csli_CIT_2g_011000 | Uncharacterized protein |
| Csli_CIT_5g_010090 | DNAJ heat shock N-terminal domain-containing protein |
| Csli_CIT_7g_000180 | E3 ubiquitin-protein ligase |
| Csli_CIT_4g_014420 | RNA polymerase-associated protein CTR9 homolog |
| Csli_CIT_1g_011450 | Belongs to the protein kinase superfamily. Ser Thr protein kinase family |
| Csli_CIT_5g_036300 | isoform X1 |
| Csli_CIT_4g_019010 | axial regulator YABBY |
| Csli_CIT_5g_021410 | disease resistance |
| Csli_CIT_1g_015200 | Belongs to the ubiquitin-conjugating enzyme family |
| Csli_CIT_4g_020660 | TOM1-like protein |
| Csli_CIT_5g_030790 | mediator of RNA polymerase II transcription subunit |
| Csli_CIT_2g_024930 | Wings apart-like protein regulation of heterochromatin |
| Csli_CIT_2g_003050 | xanthine dehydrogenase |
| Csli_CIT_4g_020630 | Prolyl oligopeptidase family |
| Csli_CIT_5g_031160 | LRR receptor-like serine threonine-protein kinase |
| Csli_CIT_3g_010630 | Belongs to the peptidase M24B family |
| Csli_CIT_5g_025900 | Belongs to the expansin family |
| Csli_CIT_6g_000100 | isoform X1 |
| Csli_CIT_2g_022650 | Uncharacterized protein |
| Csli_CIT_6g_006350 | Uncharacterized protein |
| Csli_CIT_7g_005180 | Octicosapeptide Phox Bem1p (PB1) domain-containing protein |
| Csli_CIT_1g_016190 | Uncharacterized protein |
| Csli_CIT_1g_019420 | transcription factor |
| Csli_CIT_5g_035210 | ATP synthase delta chain |
| Csli_CIT_3g_006510 | Disease resistance protein |
| Csli_CIT_2g_020730 | (E 0.0) protein kinase family protein |
| Csli_CIT_4g_014250 | isoform X1 |
| Csli_CIT_8g_000490 | transcription, DNA-templated |
| Csli_CIT_5g_006680 | Vacuolar protein sorting-associated protein |
| Csli_CIT_5g_034270 | Belongs to the SNF7 family |
| Csli_CIT_1g_016740 | Inositol hexakisphosphate |
| Csli_CIT_5g_034440 | Acyltransferase-like protein |
| Csli_CIT_2g_009130 | Uncharacterized protein |
| Csli_CIT_7g_013590 | disease resistance |
| Csli_CIT_6g_006570 | Ubiquitin-protein ligase |
| Csli_CIT_5g_014190 | leucine-rich repeat receptor-like serine threonine-protein kinase |
| Csli_CIT_3g_013560 | Beach domain-containing protein |
| Csli_CIT_1g_000960 | CSC1-like protein At4g35870 |
| Csli_CIT_2g_022640 | Uncharacterized protein |
| Csli_CIT_5g_022650 | Transcription elongation factor |
| Csli_CIT_6g_009200 | Carbon catabolite repressor protein 4 homolog |
| Csli_CIT_5g_014790 | oxidoreductase activity |
| Csli_CIT_5g_001400 | Long chain acyl-CoA synthetase |
| Csli_CIT_5g_033210 | Vacuolar protein sorting-associated protein 51 homolog |
| Csli_CIT_4g_003790 | 26S proteasome, regulatory subunit |
| Csli_CIT_7g_004060 | RNI-like superfamily protei |
| Csli_CIT_4g_011350 | Uncharacterized protein |
| Csli_CIT_2g_011540 | Uncharacterized protein |
| Csli_CIT_3g_003000 | Alpha,alpha-trehalose-phosphate synthase (UDP-forming) |
| Csli_CIT_5g_017620 | Belongs to the ubiquitin-conjugating enzyme family |
| Csli_CIT_7g_023410 | Guanylylate cyclase |
| Csli_CIT_5g_036460 | Protein of unknown function (DUF3522) |
| Csli_CIT_1g_012030 | hydrolase, alpha beta fold family protein |
| Csli_CIT_4g_019890 | glycine-rich protein |
| Csli_CIT_8g_002840 | magnesium transporter |
| Csli_CIT_1g_012990 | DUF1771 |
| Csli_CIT_8g_001160 | serine threonine-protein kinase |
| Csli_CIT_1g_020860 | Gamma-glutamyltranspeptidase |
| Csli_CIT_4g_000660 | Non-specific serine threonine protein kinase |
| Csli_CIT_5g_000430 | Uncharacterized protein |
| Csli_CIT_7g_000250 | callose synthase |
| Csli_CIT_5g_020230 | Belongs to the TRAFAC class dynamin-like GTPase superfamily. Dynamin Fzo YdjA family |
| Csli_CIT_4g_015730 | Belongs to the tetraspanin (TM4SF) family |
| Csli_CIT_5g_011900 | G patch domain-containing protein |
| Csli_CIT_7g_006720 | calmodulin-binding transcription activator |
| Csli_CIT_4g_010850 | belongs to the protein kinase superfamily |
| Csli_CIT_1g_003900 | BEST Arabidopsis thaliana protein match is LisH and RanBPM domains containing protein (TAIR AT1G61150.1) |
| Csli_CIT_5g_005870 | callose synthase |
| Csli_CIT_7g_017060 | carbohydrate kinase family protein |
| Csli_CIT_8g_009520 | calmodulin-binding transcription activator |
| Csli_CIT_8g_001690 | E3 ubiquitin-protein ligase |
| Csli_CIT_9g_008200 | Domain of unknown function (DUF3527) |
| Csli_CIT_4g_018760 | 2-alkenal reductase (NADP( )-dependent)-like |
| Csli_CIT_2g_008740 | Protein of unknown function (DUF3754) |
| Csli_CIT_9g_005990 | GDP-mannose 4,6 dehydratase |

| **Supplementary Table S3. Summary of bisulfite sequencing and alignment data for each sample.** | | | |
| --- | --- | --- | --- |
| **Variety** | **Sample** | **Raw data (Gb)** | **conversion rate (%)** |
| Eureka lemon | Fruit DAF 65-1 | 18.56 | 0.9969 |
|  | Fruit DAF 65-2 | 20.11 | 0.997 |
|  | Fruit DAF 65-3 | 18.16 | 0.9971 |
|  | Fruit DAF 135-1 | 22.43 | 0.996 |
|  | Fruit DAF 135-2 | 18.09 | 0.9965 |
|  | Fruit DAF 135-3 | 20.93 | 0.997 |
| Sweet lemon | Fruit DAF 65-1 | 18.03 | 0.9968 |
|  | Fruit DAF 65-2 | 18.56 | 0.997 |
|  | Fruit DAF 65-3 | 17.56 | 0.9971 |
|  | Fruit DAF 135-1 | 19.33 | 0.9972 |
|  | Fruit DAF 135-2 | 23.55 | 0.9968 |
|  | Fruit DAF 135-3 | 21.46 | 0.997 |

**Supplementary Table S4. Potential *CliSWEET14* regulating proteins identified by Y1H screening.**

| **Gene ID** | **Discription** |
| --- | --- |
| CsUn_pb002150.1 | NAC56 |
| CsUn_pb002440.1 | E3 ubiquitin-protein ligase RMA1H1 |
| Cs5g_pb004280.1 | Glycerophosphodiester phosphodiesterase GDPD6 |
| CsUn_pb052110.1 | ATP synthase subunit alpha, chloroplastic |

**Supplementary Table S5. List of primers or EMSA probes used in this study.**

| **Primer name** | **Primer sequence (5'-3')** |
| --- | --- |
| qPCR-Actin-F | CCGACCGTATGAGCAAGGAAA |
| qPCR-Actin-R | TTCCTGTGGACAATGGATGGA |
| qPCR-CliSWEET14-F | TCTGGGAATGGCCTTTGGAGCGAC |
| qPCR-CliSWEET14-R | ACTGTCGTGACGGTGATGATGATTGC |
| qPCR-CliNAC56-F | TGCCGACAAGGGGAGCAAA |
| qPCR-CliNAC56-R | TGATGAGCTTGAGGCAGCA |
| PDR196-SWEET-F | TCCCCCGGGCTGCAG GAATTC ATGAATCCCGATCCTGCA |
| PDR196-SWEET-R | GGGCCCCCCCTCGAG GTCGAC+ GGCGGTGTGGTTGATTTG |
| PENTR1A-CliSWEET14-F | AAAGGAACCAATTCAGTCGACATGAATCCCGATCCTGCA |
| PENTR1A-CliSWEET14-R | TGGAAAAGGGAATTCGGTACCGGCGGTGTGGTTGAGTTG |
| PENTR1A-CliNAC56-F | AAAGGAACCAATTCAGTCGACATGGAGAGTGCAGACTCATCGACG |
| PENTR1A-CliNAC56-R | TGGAAAAGGGAATTCGGTACCAGAGTACCAATTCATTCCCGGAAG |
| PENTR1A-CliABF3-F | AAAGGAACCAATTCAGTCGACATGGGATCTCAAATGAACTACAAGAACG |
| PENTR1A-CliABF3-R | TGGAAAAGGGAATTCGGTACC CCAAGGGCCTGTCAGTGTCCTCCTCAA |
| TRV2-CliSWEET14-F | AGAAGGCCTCCATGGGGATCCGCCCTAGCGAGATATTGCCGG |
| TRV2-CliSWEET14-R | TGTCTTCGGGACATGCCCGGGCGGGGGCGGTGTGGTTGAGTTGA |
| pDONR221-CliABF3-F | GGGGACAAGTTTGTACAAAAAAGCAGGCTTAATGGGATCTCAAATGA |
| pDONR221-CliABF3-R | GGGGACCACTTTGTACAAGAAAGCTGGGTTCCAAGGGCCTGTCAGTG |
| Chop–PCR-CliSWEET14-F | AGAAGGTAACTCATTAGCTAGCTTGTC |
| Chop–PCR-CliSWEET14-R | GAGAATTGACACCCATTATTAGGTAGTCG |
| BS-PCR-CliSWEET14-F | AGAAGGTAATTTATTAGTTAGTTTGTTAATATTG |
| BS-PCR-CliSWEET14-R | TTGGTTGTTTTTTATTATCCTTAACACTTAATAC |
| 3C-a | TGCCTAGATAGACCATTTTCC |
| 3C-b | TCTGAACTATGAATGGGGCGGCCG |
| 3C-c | CCTCGTTGCCGTCGCATTCA |
| 3C-d | TGCTAGGACTGGGATGGGTCC |
| 3C-e | CACCTGCATTTTGAATTTGTTTGG |
| 3C-f | TAGGTCGGCATTGGTCTAGC |
| 3C-g | TGTTTTGGTTCGATCGGGTTACG |
| pAbAi-CliSWEET14(3′-UTR)-F | CTTGAATTCGAGCTC GGTACC TGCAGGTGAAAATTTATCGTATGCAC |
| pAbAi-CliSWEET14(3′-UTR)-R | ATACAGAGCACATGC CTCGAG TCCGGATTGAGAGTTATCGTGCATAC |
| pGADT7-ClibHLH105-F | GATTACGCTCATATG ATGGAGATTGCGTCATCTGACAACTC |
| pGADT7-ClibHLH105-R | ACCCGGGTGGAATTC AGCAACCGGAGGACGAAGTACGTGATCC |
| LUC-CliSWEET14(3′-UTR)-0800-F | GTCGACGGTATCGATAAGCTT TGCAGGTGAAAATTTATCGTATGCAC |
| LUC-CliSWEET14(3′-UTR)-0800-R | CGCTCTAGAACTAGTGGATCC TCCGGATTGAGAGTTATCGTGCATAC |
| LUC-ClibHLH105-F | CGCTCTAGAACTAGTGGATCC ATGGAGATTGCGTCATCTGACAACTC |
| LUC-ClibHLH105-R | GATAAGCTTGATATCGAATTC AGCAACCGGAGGACGAAGTACGTGATCC |
| pAbAi-SWEET14-F | CTTGAATTCGAGCTC GGTACC CTCTGCCTAGATTTTTAACATTCAC |
| pAbAi-SWEET14-R | TACAGAGCACATGC CTCGAG TTGAAAATTAATAAGCTCTCTCTAGC |
| pAbAi-CliNAC56-F | CTTGAATTCGAGCTCGGTACC AAGGGTGTCGTGTACTGGGGGCA |
| pAbAi-CliNAC56-R | TACAGAGCACATGCCTCGAGCTAGCTAGCTGCAATTAACCCAAGATC |
| pGADT7-CliNAC56-F | GATTACGCTCATATG ATGGAGAGTGCAGACTCATCGACG |
| pGADT7-CliNAC56-R | ACCCGGGTGGAATTC AGAGTACCAATTCATTCCCGGAAG |
| pGADT7-CliABF3-F | GATTACGCTCATATG+ATGGGATCTCAAATGAACTACAAGAACG |
| pGADT7-CliABF3-R | ACCCGGGTGGAATTC+CCAAGGGCCTGTCAGTGTCCTCCTCAA |
| Probe (For CliSWEET14) | GGGGTTTCATTTACATCACCATCACGTTTTAAATCAAGCCTCTGGCC |
| mProbe(For CliSWEET14) | GGGGTTTCATTTACATCACCATTTTTTTTTAAATCAAGCCTCTGGCC |
| Probe1 (For CliNAC56) | GGTGCCCACCACTGTCCCCACTAACGTGTCACCCTCTCATTTCTCTC |
| mProbe1 (For CliNAC56) | GGTGCCCACCACTGTCCCCACTCCCCCCCCACCCTCTCATTTCTCTC |
| Probe2 (For CliNAC56) | CCGTTTAGAGGACGACACGTGTAATTCGGAGAGAAATGAG |
| mProbe2 (For CliNAC56) | CCGTTTAGAGGACGACCCCCCCAATTCGGAGAGAAATGAG |
| Probe3 (For CliNAC56) | CTAATTAGCCCTTATTTCCATTACGTGTCGACACAACATTAGTC |
| mProbe3 (For CliNAC56) | CTAATTAGCCCTTATTTCCATCCCCCCCCGACACAACATTAGTC |
| LUC-CliSWEET14-0800-F | GTCGACGGTATCGATAAGCTT CTCTGCCTAGATTTTTAACATTCAC |
| LUC-CliSWEET14-0800-R | CGCTCTAGAACTAGTGGATCC TTGAAAATTAATAAGCTCTCTCTAGC |
| LUC-CliNAC56-0800-F | GTCGACGGTATCGATAAGCTT AAGGGTGTCGTGTACTGGGGGCA |
| LUC-CliNAC56-0800-R | CGCTCTAGAACTAGTGGATCCCTAGCTAGCTGCAATTAACCCAAGATC |
| 62-SK-CliNAC56-F | CGCTCTAGAACTAGTGGATCC ATGGAGAGTGCAGACTCATCGACG |
| 62-SK-CliNAC56-R | GATAAGCTTGATATCGAATTC AGAGTACCAATTCATTCCCGGAAG |
| 62-SK-CliABF3-F | CGCTCTAGAACTAGTGGATCC ATGGGATCTCAAATGAACTACAAGAACG |
| 62-SK-CliABF3-R | GATAAGCTTGATATCGAATTC CCAAGGGCCTGTCAGTGTCCTCCTCAA |
| F1 fragment-F (For CliNAC56) | TCTGCAAACAAGAGGGATTGC |
| F1 fragment-R (For CliNAC56) | CCCCAGTACACGACACCCTT |
| F2 fragment-F (For CliNAC56) | TGAAGAGAGTATCTTGATCCCTG |
| F2 fragment-R (For CliNAC56) | GCGTAGCGTCTCACTATCAA |
| F3 fragment-F (For CliNAC56) | TTGTGGTGCCCACCACTGTCCCCAC |
| F3 fragment-R (For CliNAC56) | TCGGAGAGAAATGAGAGGGTGA |
| F4 fragment-F (For CliNAC56) | ACCCTCTCATTTCTCTCCGA |
| F4 fragment-R (For CliNAC56) | CGGCCCGTGGTGCCGT |
| F1 fragment-F (For CliNAC56) | TGCATGGGGATACAACA |
| F1 fragment-R (For CliNAC56) | ATGTGGTGGCAGGGTGGAA |
| DX2181-pSWEET14-F (PstI) | CTACAGCGCTAAGCTTGGCTGCAG CTCTGCCTAGATTTTTAACATTCAC |
| DX2181-pSWEET14-R (BamHI) | AAGGGACTGACCACCCGGGATCCTTGAAAATTAATAAGCTCTCTCTAGC |
| DX2181-pNAC56-F (PstI) | CTACAGCGCTAAGCTTGGCTGCAG AAGGGTGTCGTGTACTGGGGGCA |
| DX2181-pNAC56-R (BamHI) | AAGGGACTGACCACCCGGGATCC CTAGCTAGCTGCAATTAACCCAAGATC |
| YFP-CliSWEET14-F | ATGGGATCTACTAGTGAATTCATGAATCCCGATCCTGCA |
| YFP-CliSWEET14-R | GGGGGTACCGTCGACGGATCCGGCGGTGTGGTTGATTTG |
| YFP-CliNAC56 | ATGGGATCTACTAGTGAATTCATGGAGAGTGCAGACTCATCGACG |
| YFP-CliNAC56-R | GGGGGTACCGTCGACGGATCCAGAGTACCAATTCATTCCCGGAAG |
| YFP-CliABF3-F | ATGGGATCTACTAGTGAATTC ATGGGATCTCAAATGAACTACAAGAAC |
| YFP-CliABF3 | GGGGGTACCGTCGACGGATCCCCAAGGGCCTGTCAGTGTCCTCCTCA |
| JW771-CliABF3-F | ACGGGGGACGAGCTCGGTACCATGGGATCTCAAATGAACTACAAGAAC |
| JW771-CliABF3 | CGCGTACGAGATCTGGTCGACCCAAGGGCCTGTCAGTGTCCTCCTCA |
| JW772-CliNAC56-F | TACGCGTCCCGGGGCGGTACC ATGGAGAGTGCAGACTCATCGACG |
| JW772-CliNAC56-R | ACGAAAGCTCTGCAGGTCGAC AGAGTACCAATTCATTCCCGGAAG |
| YNE-CliNAC56-F | TGGCGCGCCACTAGTGGATCCATGGAGAGTGCAGACTCATCGACG |
| YNE-CliNAC56-R | AGCGGTACCCTCGAGGTCGACAGAGTACCAATTCATTCCCGGAAG |
| YCE-CliABF3-F | TGGCGCGCCACTAGTGGATCCATGGGATCTCAAATGAACTACAAGAAC |
| YCE-CliABF3-R | AGCGGTACCCTCGAGGTCGACCCAAGGGCCTGTCAGTGTCCTCCTCAAG |
